# Viscosity as a Smoking Gun for Complex Formation in Solution: Fe^2+^ and Mg^2+^ Chlorides as Examples

**DOI:** 10.1021/acs.jpcb.6c01329

**Published:** 2026-03-16

**Authors:** Amrita Goswami, Samuel Blazquez, Lucía Fernández-Sedano Vázquez, Eva González Noya, Hannes Jónsson, Jacobo Troncoso, Carlos Vega

**Affiliations:** † Science Institute and Faculty of Physical Sciences, 63541 University of Iceland, VR-III 107 Reykjavík, Iceland; ‡ Departamento de Química Física, Facultad de Ciencias Químicas, 16734 Universidad Complutense de Madrid, 28040 Madrid, Spain; § 16379 Instituto de Química Física Blas Cabrera, CSIC, C/Serrano 119, 28006 Madrid, Spain; ∥ Departamento de Física Aplicada, Universidade de Vigo, Escola de Enxeñaría Aeronaútica e do Espazo, E 32004 Ourense, Spain

## Abstract

Electrolyte solutions at high concentration are indispensable and yet poorly understood. In particular, the extent of speciation—the formation of complexes composed of multiple species—in concentrated ionic solutions is very challenging to obtain theoretically and experimentally, but can have a strong effect on solution properties. The literature is rife with contradictory estimates of speciation from experiments. We find that speciation affects transport properties and is therefore a prerequisite to accurately model concentrated solutions. We turn this to our advantage by showing that the viscosity can be used to determine the extent of complexation in concentrated aqueous solutions. Results of simulations as well as experimental measurements are presented. The atomistic Madrid-2019 force field is extended to model FeCl_2_. Solutions of FeCl_2_ and MgCl_2_ are compared, and the observed difference in viscosity is explained by more complexation in the former, a conclusion supported by recently reported X-ray absorption and neutron scattering experiments.

## Graphical abstract

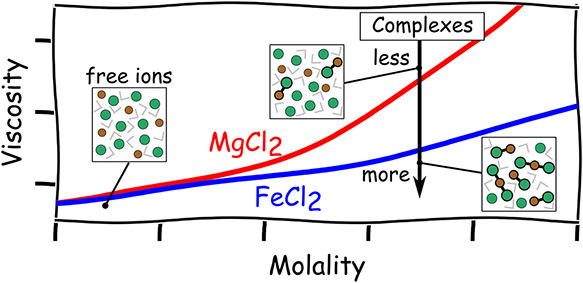

## 1 Introduction

Electrolytes in water are omnipresent in nature and play a vital role in practical and industrial applications.^1–4^ For example, seawater is an electrolyte solution,^5^ and many of the ions that are abundant in the sea are also present in living cells.^6^ Given the ubiquity and relevance of electrolyte solutions, it is perhaps surprising that a rigorous and practical theory for electrolytes at high concentration is not available. Many successful theories, such as the elegant Debye–Hückel theory,^7^ approach electrolyte solutions from the infinitely dilute limit, and thus, quite intuitively, work only for dilute solutions.^8^ The behavior of electrolyte solutions at high concentration is difficult to extrapolate from this infinitely dilute limit because of effects such as complex formation, ion pairing, etc., which cannot be easily shoehorned into a single mathematical framework.

The extent to which ions of opposite charge form long-lived ion pairs is, therefore, an important and divisive issue. The existence of complexes can significantly affect various properties of electrolyte solutions, particularly so at high concentration.^9^ However, experimental estimates differ widely on the extent of complexation, as well as on the equilibrium distribution of different types of complexes. For example, values reported in the literature for the equilibrium constant, log *K*, of FeCl^+^ formation in aqueous FeCl_2_ solution, range from 0.74 ^10^ to −0.89,^11^ with multiple values in between.^12–17^ Various measurements at 4 m have, in particular, yielded contradictory results. The spectrophotometric experiments of Zhao and Pan^17^ suggest that the monochloro complex [FeCl­(H_2_O)_5_]^+^ is the predominant species in solution, with a small amount of the neutral trans dichloro complex [FeCl_2_(H_2_O)_4_]. However, X-ray absorption spectroscopy measurements of Luin et al.^18^ provide evidence for the conclusion that the neutral trans dichloro complex [FeCl_2_(H_2_O)_4_] is the dominant species. In contrast, THz/FIR absorption spectra of Böhm et al.^11^ indicate the presence of only the monochloro complex.

Chemical equilibrium models can predict speciation using activity models, for example Specific Ion Theory (SIT)^19–21^ and the Davies equation,^22^ using ion association constants (typically obtained from experiments).^13,23^ However, the quality of the results depends strongly on (i) the activity model used, which may fail at high concentration, and (ii) the accuracy of the ion association constants. Discrepancies can arise due to inherent differences in experimental measurement methods. For instance, an equilibrium constant calculated using the anion exchange method,^24^ which is sensitive only to contact ion pairs, can be smaller than one estimated using potentiometry.^25^

Atomic-scale simulations using empirical potential functions can only approximately describe the interaction between molecules and ions. Despite this, they can still be used to learn some physics about electrolytes, thus extending our understanding of ionic solutions (at least qualitatively) beyond the Debye–Hückel limit. However, predicting both the dynamics of complex formation and the equilibrium distribution of complexes accurately can be challenging with simple point charge models.^26^

Density functional theory (DFT) has been successfully used to describe interactions between cations, water and anions in ionic solutions.^27–29^ However, although quantum mechanical calculations can provide insight into the stability of specific complexes, they are infeasible to determine the equilibrium distribution of complexes due to their high computational costs and limited system size.

Therefore, the degree of complexation is difficult to ascertain from experiments, simulations and theory. On the other hand, as discussed above, knowledge of complexation and complex distributions is crucial, since they can strongly affect solution properties. Here, we demonstrate that this problem can be turned on its head: viscosity can instead be used as a guide to infer the extent of complexation.

We accomplish this by performing both experiments, for FeCl_2_, and simulations, comparing FeCl_2_ and MgCl_2_. In order to describe FeCl_2_ in simulations, we extend the Madrid-2019 force-field,^30^ which is a simple empirical force-field wherein monatomic ions are represented by single Lennard-Jones (LJ) centers with a scaled charge. We include complexes in our simulations by freezing distances between cation-chloride ion pairs. By explicitly including a fixed number of monochloro and dichloro complexes, and varying these constant proportions over a number of simulation trajectories, we show that the effect on the viscosity is surprisingly large and measurable. We present clear evidence of higher association in FeCl_2_, compared to MgCl_2_. This is in contrast with the equilibrium values for association recommended by NIST,^31^ but which is in line with more recent experimental measurements.^18,32^

## 2 Methods

### 2.1 Experimental Section

Ferrous chloride was purchased from Sigma-Aldrich, with a purity greater than 0.99 in mass fraction. Milli-Q water was used to prepare the solutions, made by weight in an AE-240 Mettler Toledo balance, with an uncertainty of 0.1 mg, and in an atmosphere of N_2_ to avoid Fe^2+^ oxidation. Uncertainty in concentration is estimated to be 0.004 mol/kg. Densities were measured using a DMA5000 vibrating tube densimeter from Anton Paar. Since the densities of the investigated solutions reach high values, calibration was performed using Milli-Q water and carbon tetrachloroethylene, purchased from Sigma-Aldrich with a purity greater than 0.999 in mass fraction. The viscosity of the solutions is small enough to neglect any correction due to damping of the vibrating tube oscillations. Uncertainty in density is estimated to be 0.0003 g/cm^3^. More details about the procedure for density measurements can be found elsewhere.^33^ Viscosities were measured using an AMVn falling ball viscometer, calibrated using Milli-Q water, details of which are present in the literature.^34^ Relative uncertainty in this magnitude is estimated to be 2%. The experimental technique was validated by reproducing experimental densities and viscosities of MgCl_2_ from previous work.^35,36^ The density and viscosity were determined for 14 solutions at 298.15 K, in the concentration interval (0–4.1) mol/kg.

### 2.2 Computational

Molecular Dynamics (MD) simulations were performed using both GROMACS (version 4.6.7 and 2015.2)^37^ and LAMMPS (29 Aug 2024, Update 1);^38^ see Sections 1.1.1 and 1.1.2 in the SI for details. All results were obtained at ambient conditions (i.e., 298.15 K and 1 bar). Atomic-scale simulations have been performed using the Madrid-2019 force-field,^30^ which combines the TIP4*P*/2005 model of water^39^ and scaled charges for the ions, such that monatomic cations have a charge of *q* = 0.85 *e*. The use of scaled charges (also denoted as the Electronic Continuum Correction) is becoming a popular strategy in the literature.^8,40–46^

A version of the force-field with a charge of 0.80 *e* has also been used in this work. Parameters for Mg^2+^ (for *q* = 0.85 *e*) and Cl^–^ (for both *q* = 0.85 *e* and 0.80 *e*) have been reported previously^30,47^ whereas the parameters for Mg^2+^ (for *q* = 0.80 *e*) were obtained in this work. The proposed force-field parameters for Fe^2+^ and Cl^–^ are presented in Table 1. These parameters are the same as those of Mg^2+^.^30^ Justifications for using the same force-field are provided in Section 3.2.1. Naturally, the correct masses should be used for Fe^2+^ and Mg^2+^.

**Table 1:** Force-Field Parameters for the Fe^2+^ and Cl^–^ Ions^a^

	*q* = 0.85 *e*	
	σ_*ij*_ (nm)	ϵ_*ij*_ (kJ/mol)
Fe^2+^–Fe^2+^	0.116290	3.651900
Fe^2+^–Cl^–^	0.300000	3.000000
Fe^2+^–O_w_	0.181000	12.00000
Cl^–^–Cl^–^	0.469906	0.076923
Cl^–^–O_w_	0.423867	0.061983

	*q* = 0.80 *e*	
	σ_*ij*_ (nm)	ϵ_*ij*_ (kJ/mol)
Fe^2+^–Fe^2+^	0.116290	3.651900
Fe^2+^–Cl^–^	0.300000	3.000000
Fe^2+^–O_w_	**0.185000**	12.00000
Cl^–^–Cl^–^	0.469906	0.076923
Cl^–^–O_w_	**0.418801**	0.061983

a The values for *q* = 0.85 *e* are those of the Madrid-2019 force field for Mg^2+^. The parameters which are different in the *q* = 0.80 *e* model, compared to the original model with *q* = 0.85 *e*, are highlighted in bold text. The molality scale, *m* (moles of salt per kg of solution), has been used for concentrations.

## 3 Results

### 3.1 Measured Density and Viscosity of FeCl_2_

Although the solubility limit of FeCl_2_ is about 5.5 m at room temperature and ambient pressure,^48^ the literature lacks values for the density and viscosity for concentrations above 2 m^35^ and 0.4 m,^36^ respectively. Data is reported here for up to 4 m. Interpolated values at round numbers for the density and viscosity are provided in Table 2. Good agreement is found with existing literature values for the more dilute solutions [see also Figure S2a in the SI].^35,36^

**Table 2:** Experimentally Measured Density and Viscosity of Aqueous Solutions of FeCl_2_ at Various Concentrations

molality (mol/kg)	density (g/cm^3^)	η (mPa·s)
1.000	1.10188	1.286
2.000	1.19758	1.846
3.000	1.28578	2.663
4.000	1.36809	4.170

Figure 1 shows the experimental viscosities of FeCl_2_ and MgCl_2_ at both low and high concentration. For concentrations less than 2 m, the viscosities are similar, within errors (lower inset in Figure 1). The viscosity of electrolyte solutions is often described by the Jones-Dole equation:^49^

1 $$\eta /\eta_{w}=1+A\sqrt{m}+\mathrm{Bm}+Cm^{2}$$

where η is the viscosity of the aqueous solution, η_*w*_ is the viscosity of water, *m* is the molality, the term *A* [which is small^50^ and around 0.02 (kg/mol)^1/2^ for MgCl_2_ and FeCl_2_] describes charge–charge contributions in the highly diluted region. The *B* coefficient (in units of kg/mol) is characteristic of individual ions, is additive, and can be interpreted in terms of ion–water interactions.^51^ The term *C* [in units of (kg/mol)^2^] is thought to depend on solute–solute association effects.^52^

**Figure 1:**
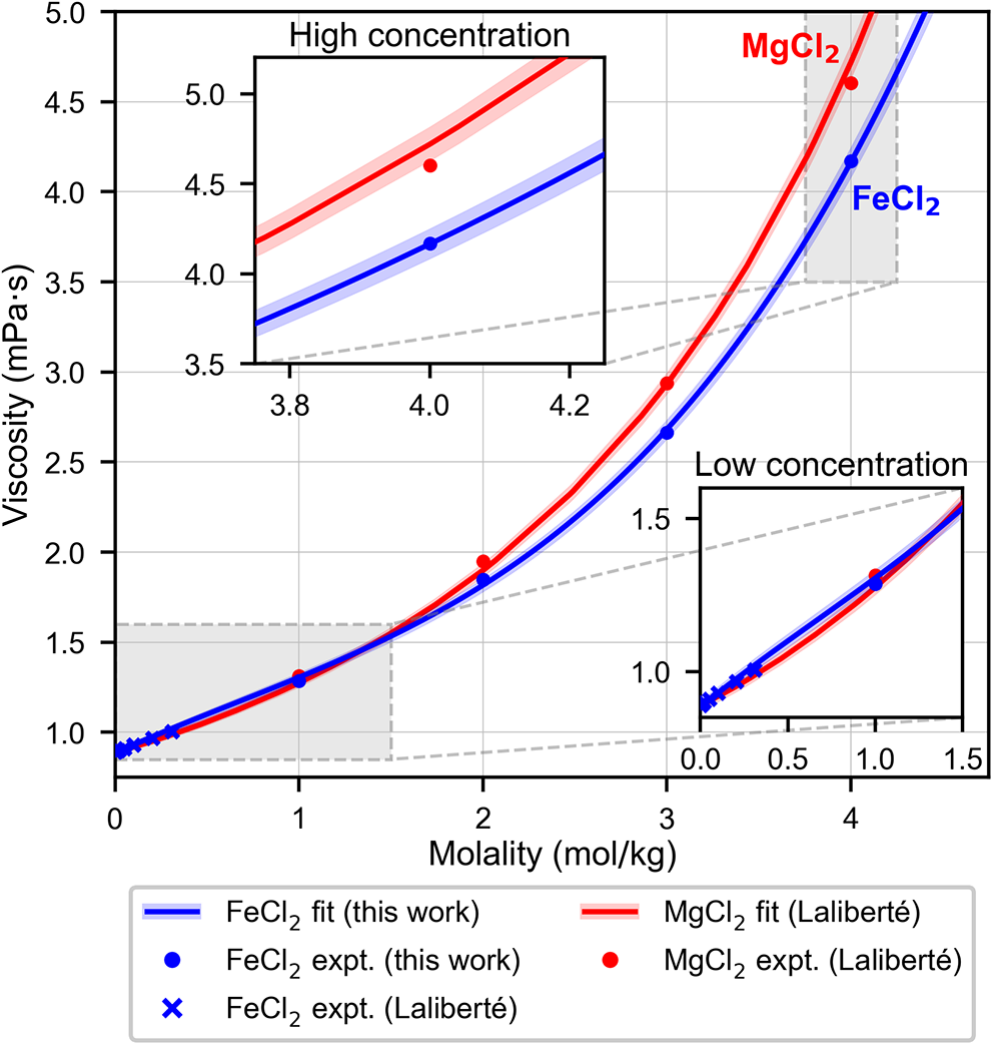
Viscosity of FeCl_2_ and MgCl_2_ from experiments, obtained from Laliberté^36^ and this work. The blue shaded region corresponds to a 2% relative uncertainty in the FeCl_2_ viscosity measurements reported in this work. The red shaded region represents the 1.8% mean relative deviation of the Laliberté model for MgCl_2_ from the experimental data.^36^ Inset, bottom: Zoomed-in view of viscosities at low concentration, which are similar in value. Inset, top: Close-up view of the stark difference between the experimental viscosity of FeCl_2_ and MgCl_2_ at high concentration.

Typically in experiments, the Jones-Dole coefficient *B* is obtained by fitting the viscosity at low concentrations (i.e., below 0.6 m), where the quadratic term can be safely neglected. As can be expected from the similar viscosities of FeCl_2_ and MgCl_2_ at low concentration, the experimental values of *B* for FeCl_2_ and MgCl_2_ are almost identical, equal to 0.405(10) kg/mol and 0.375(10) kg/mol, respectively (see also Section 3.2.2 for a comparison with simulations).^49^ The value of *B* for FeCl_2_ is slightly higher than that of MgCl_2_, which could account for the corresponding marginally higher viscosity of the former compared to the latter at low concentration.

However, the experimental viscosities of FeCl_2_ and MgCl_2_, at high concentration, differ significantly, which is highlighted in the top inset of Figure 1. Equation 1 describes the viscosities well up to a concentration of 3 m. We have estimated the values of *C* as 0.12 (kg/mol)^2^ and 0.08 (kg/mol)^2^ for MgCl_2_ and FeCl_2_, respectively, by fitting the experimental viscosities up to 3 m. At higher concentrations, a cubic *D* term [with units of (kg/mol)^3^] would have to be added to eq 1 to reproduce the experimental viscosities.

The existence of monochloro and dichloro complexes in FeCl_2_ has been reported at concentrations around 4 m.^11,17,18^ On the other hand, MgCl_2_ being a strong 2:1 electrolyte, reportedly shows little or no complexation even at higher concentrations.^26,53,54^ In subsequent sections, we provide evidence from simulations that suggests that the difference between Fe^2+^ and Mg^2+^ in their propensity to complexation is tied to differences in their viscosity at high concentration, as already suggested by the large difference in the value of *C* (33%) between both systems.

### 3.2 Simulations without Complexes

#### 3.2.1 A Force-Field for FeCl_2_

Figure 2 presents a comparison of experimental densities with those obtained from simulations, using the same force-field for MgCl_2_ and FeCl_2_, at various concentrations. Agreement between simulations and experiments is within 0.5% for both the 0.85 and 0.8 charge models. Solution densities are often used as target properties in parametrization,^30,55^ and consequently, the good agreement with experiments at low and high concentration validates the approximation of using Mg^2+^ force-field parameters for Fe^2+^.

**Figure 2:**
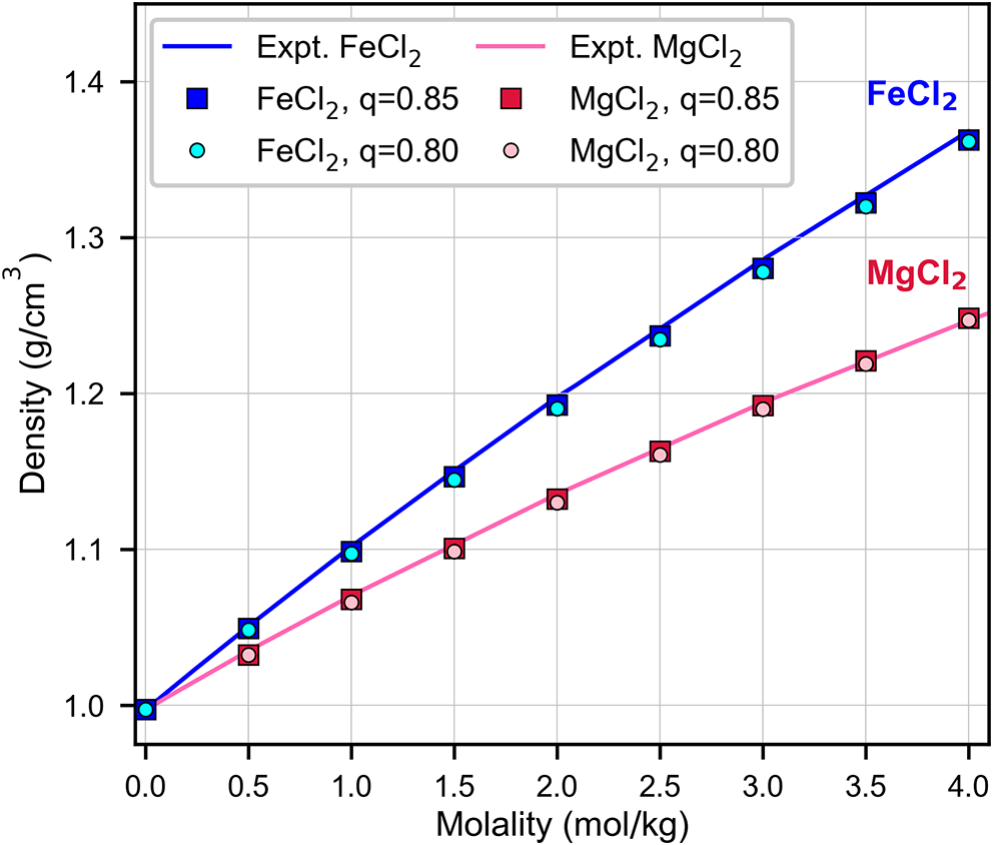
Density of FeCl_2_ and MgCl_2_ from fits to experiments (solid blue and magenta line, respectively) from this work and Cooper,^35^ Laliberté,^36^ respectively, as well as the density from atomic-scale simulations without complexes (squares and circles). Agreement is within 0.5% for all concentrations.

When the same force-field is used for FeCl_2_ and MgCl_2_, an inherent assumption is that the number densities of both solutions are the same and that, consequently, the following relation holds true:

2 $$\rho_{{\mathrm{FeCl}}_{2}}=\rho_{{\mathrm{MgCl}}_{2}}\frac{1000+m\times M_{{\mathrm{FeCl}}_{2}}}{1000+m\times M_{{\mathrm{MgCl}}_{2}}}$$

where ρ_MgCl_2__ and ρ_FeCl_2__ are the mass densities in g/cm^3^, *M*_MgCl_2__ and *M*_FeCl_2__ are the molar masses in g/mol of MgCl_2_ and FeCl_2_, respectively, and *m* is the molality of the solutions in mol/kg.

At low concentration, the assumption of equal number densities is a reasonable one, since the experimental values of the cation-oxygen distance and the hydration free energy of Fe^2+^ and Mg^2+^ are quite similar (collected in Table S1 in the SI). In order to evaluate whether this assumption is also valid at higher concentrations, we estimated the mass density of FeCl_2_, using eq 2, from experimental MgCl_2_ densities, and compared the former with corresponding experimental densities, obtaining good agreement, as shown in Figure S2a in the SI. This simple test is also passed for Fe­(SO_4_) and Mg­(SO_4_), as shown in Figure S2b in the SI. More generally, we believe that eq 2 can be used to easily test whether the force-field parameters of one cation can be reasonably used for another target cation (at least for density predictions) since such an evaluation would only require experimental densities at different concentrations.

#### 3.2.2 Transport Properties of Solutions without Complexes

The experimental data obtained for FeCl_2_ enables us to examine trends in the viscosity at high concentration, where the effect of complexes is expected to be important. However, complexes do not form spontaneously in our atomic-scale simulations of FeCl_2_ and MgCl_2_. This is because obtaining the equilibrium complex population would require much longer simulation time, on the order of microseconds, since this is the experimental residence time of water^56,57^ in solvation shells of Mg^2+^ or Fe^2+^.

Figure 3 shows the experimental values for the viscosity of FeCl_2_ and MgCl_2_, along with results obtained from simulations (red and blue squares). At concentrations below 2 m, the viscosity of MgCl_2_ and FeCl_2_ obtained from simulations agree with experiments well, and this is also the regime where the experimental viscosities are quite similar (lower inset in Figure 1).

**Figure 3:**
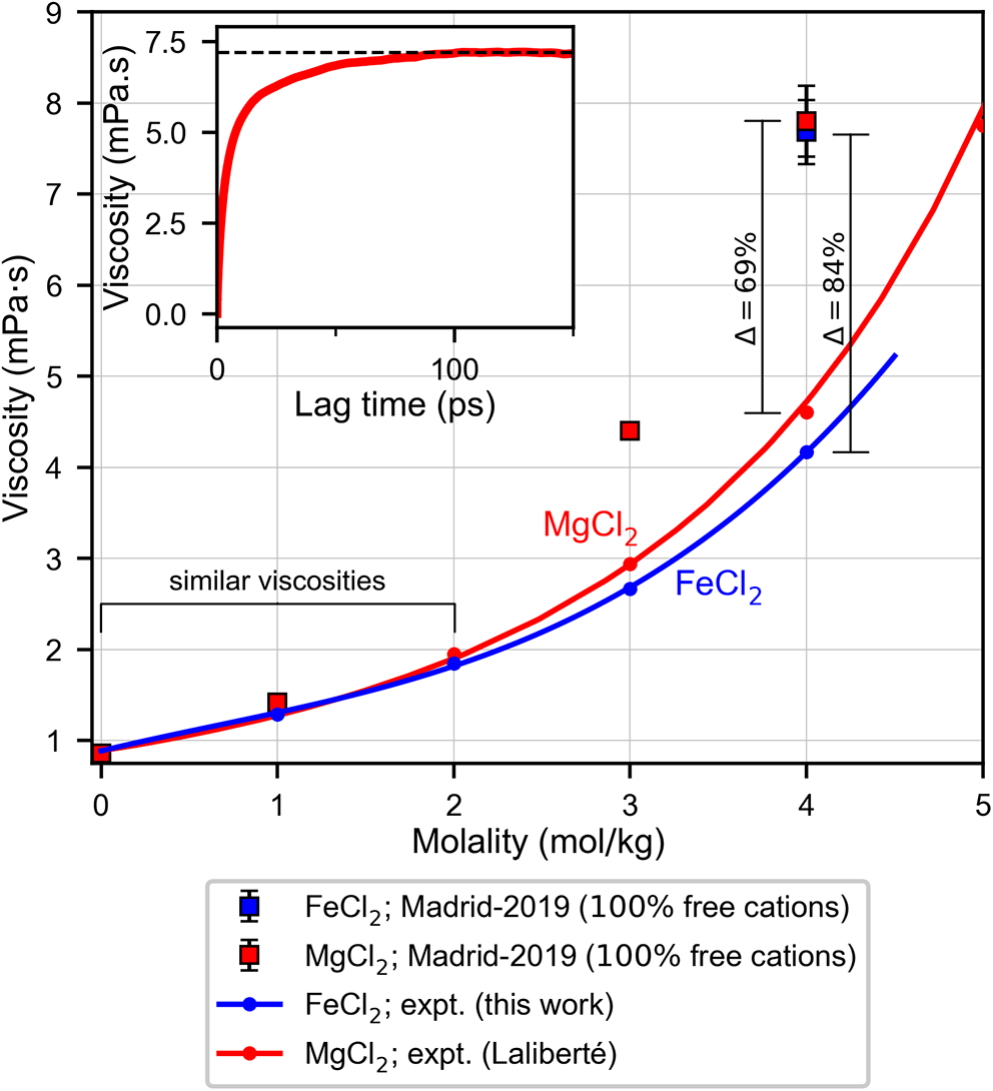
Comparison of the viscosity of FeCl_2_ and MgCl_2_ obtained from simulations using the Madrid-2019 model (blue and red squares) with those from experiments (solid blue and red line, respectively). Inset: The viscosity obtained from the Green–Kubo formalism for a 4 m MgCl_2_ solution, showing that the viscosity plateaus at about 100 ps at the converged value.

Furthermore, at a concentration of 0.6 m, it can be assumed that the quadratic term in eq 1 is negligible (elaborated in Section S12 in the SI). We estimated *B* coefficients for the 0.80 Madrid-2019 model by determining the viscosity at this concentration (see also Section S12 in the SI for details of these calculations). Table 3 presents these values, which are about 0.04 kg/mol higher than experimental values. Given that nonpolarizable models with unscaled charges are known to overestimate *B* by approximately 0.16 kg mol^–1^ for 1:1 electrolytes,^58^ with even larger deviations expected for 2:1 electrolytes, the level of agreement observed here is very good.

**Table 3:** Jones-Dole *B* Coefficients from Simulations (*B*_sim_), Computed Using the 0.80 Madrid-2019 Model, and Those from Experiments (*B*_expt_) for FeCl_2_ and MgCl_2_

system	*B*_sim_ (kg/mol)	*B*_expt_ (kg/mol)
FeCl_2_	0.46(3)	0.405(10)^a^
MgCl_2_	0.42(3)	0.375(10)^a^

a From Marcus.^49^

On the other hand, Figure 3 shows that results from simulations deviate significantly from experiments at high concentrations around 4 m. The trends in Figure 3 seem to suggest that the microstructure of FeCl_2_ and MgCl_2_ solutions is inherently different at high concentration, and that simulations which do not account for this fail to describe such solutions.

### 3.3 Simulations with Complexes

As touched upon previously in Section 3.2.2, the formation of complexes is not observed even after 200 ns runs with the Madrid-2019 force-field. Additional interactions would need to be included to capture complex formation.^26^ We conclude that both the long time scale for attaining the equilibrium complex distribution in concentrated solutions, and the challenge of describing the interaction between the ions at such close range, make simulations describing the dynamics of complex formation impractical at this time (see Section 4.3 for an expanded discussion).

Here, we make a distinction between the somewhat synonymous terms – contact ion pairs (CIPs) and complexes – used in the literature. We use the term CIP when the residence time of the anion in contact with the cation is relatively low (from a few to dozens of picoseconds), such as in the case of NaCl.^59^ We assume that the lifetime of such CIPs is less than the typical decay time of the stress autocorrelation function for the solution (which is around 100–200 ps for the systems considered in this work as shown in the inset of Figure 3). In this work, we use the term complex when the residence time of the anion in contact with the cation is significantly larger than the decay time of the stress autocorrelation function, so that we can assume that their distribution does not change significantly during the course of a simulation trajectory used to calculate physical quantities of interest (here, the viscosity and diffusion coefficient).

#### 3.3.1 A Simulation Strategy for Including Complexes

In our simulations, we model complexes by freezing the cation-chloride distance to 2.33 Å (see Section 4 of the SI for justifications) Concomitantly, water molecules are allowed to move, and are free to translate, rotate, and leave the solvation shell. Freezing the cation–anion distances is a reasonable approximation, since cation-chloride vibrations within the complex are not expected to have a significant effect on the viscosity or diffusion coefficient.

Complexes can also exhibit a plethora of structures, which is why speciation generally refers to a distribution of these types. We use the following terminology to describe two of the most dominant types of complexes formed by Fe^2+^ ^11,17,18^ and Mg^2+^,^54,60^ illustrated in Figure 4. A *monomer* (Figure 4 left) refers to a complex formed by one cation, coordinated with one Cl^–^ anion and some additional molecules of water in the first solvation shell, with the cation-chloride distance frozen. A *dimer* (Figure 4 right) refers to a complex in which there are two Cl^–^ anions in the trans position of the octahedron, along with additional coordinating water molecules.

**Figure 4:**
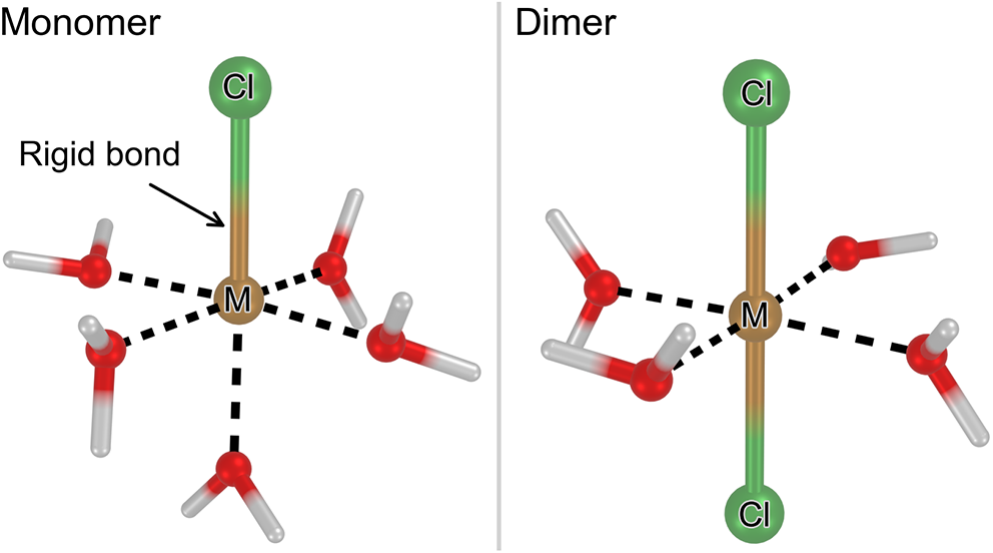
Illustration of the complexes considered in the atomic-scale simulations. The bond between the metal cation and Cl^–^ anion is rigid, but the water molecules are free to move. The black dashed lines indicate the octahedral shape of the solvation shell. *Left:* The *monomer* complex, wherein the cation is part of a monochloro complex (where M corresponds to an Fe^2+^ or Mg^2+^ cation). *Right:* The *dimer* complex, consisting of a linear dichloro unit Cl–M–Cl.

In our new simulation strategy, instead of attempting to model the dynamics of complex formation, we *introduce a fixed number of complexes* (monomers and/or dimers) at the beginning of simulations as an input and constrain the cation-chloride distance in the complex to study the effect that complex distributions can have on the properties of the solutions.

This strategy was validated by analyzing the structures of the solvation shell around free cations, monomers and dimers. The octahedral solvation structure was retained by monomers and dimers, showing that constraining the cation-chloride distance does not distort the solvation shell around the cation (Figure S3 in the SI).

In addition, the effect of complexes on the density is small, at least when considering only octahedral complexes (see Table S3 in SI; the density increases by only 0.5% at 4 m when all cations participate in monomers and there are no free cations). Therefore, the introduction of monomers and dimers does not cause the excellent density predictions of the force-field to deteriorate.

#### 3.3.2 The Effect of Complexes on Transport Properties

We denote α as the percentage of free cations, α′ as the percentage of cations in monomers and α″ as the percentage of cations in dimers, such that

3 $$\alpha +\alpha^{\prime }+\alpha^{\prime \prime }=100$$

First, we analyze the impact of monomers (illustrated in Figure 4) on the viscosity. When considering only free ions and monomers, α″ is zero. We vary the fraction of free cations, α, from 100% (where all ions are free) to 0% (where all cations form monomers).

The viscosity and diffusion coefficient of water obtained in simulations for 4 m solutions of FeCl_2_ and MgCl_2_ are presented in Table 4. Figure 5 shows the viscosities of MgCl_2_ and FeCl_2_ at 4 m (at 298.15 K and 1 bar), for different complex distributions (i.e., different proportions of free cations and monomers). The experimental viscosities of MgCl_2_ and FeCl_2_ at this concentration are also depicted. We observe that the viscosities of FeCl_2_ and MgCl_2_ from simulations are roughly the same (within our uncertainty, which is 0.25–0.45 mPa·s). Notably, the presence of complexes (monomers, in this case) significantly reduces the value of the viscosity – the viscosity of MgCl_2_ goes from ≈ 7.73 mPa·s when α = 100 to ≈5.5 mPa·s when α = 0. Note that this last value of the viscosity, 5.5 mPa·s, is closer to the experimental result of MgCl_2_ (4.73 mPa·s).^36^

**Table 4:** Viscosity (in mPa·s) of FeCl_2_ and MgCl_2_ Solutions at 4 m Obtained from Simulations Using the Madrid-2019 Force Field^a^

system	α	*D*_H_2_O_ × 10^9^ (m^2^/s)	η (mPa·s)
MgCl_2_	100	0.366	7.73(0.39)
MgCl_2_	15	0.499	5.71(0.33)
MgCl_2_	2.5	0.522	5.56(0.42)
FeCl_2_	100	0.369	7.68(0.41)
FeCl_2_	15	0.496	5.87(0.30)
FeCl_2_	2.5	0.522	5.54(0.32)

a The Yeh-Hummmer correction^61^ was used to account for finite-size effects.

**Figure 5:**
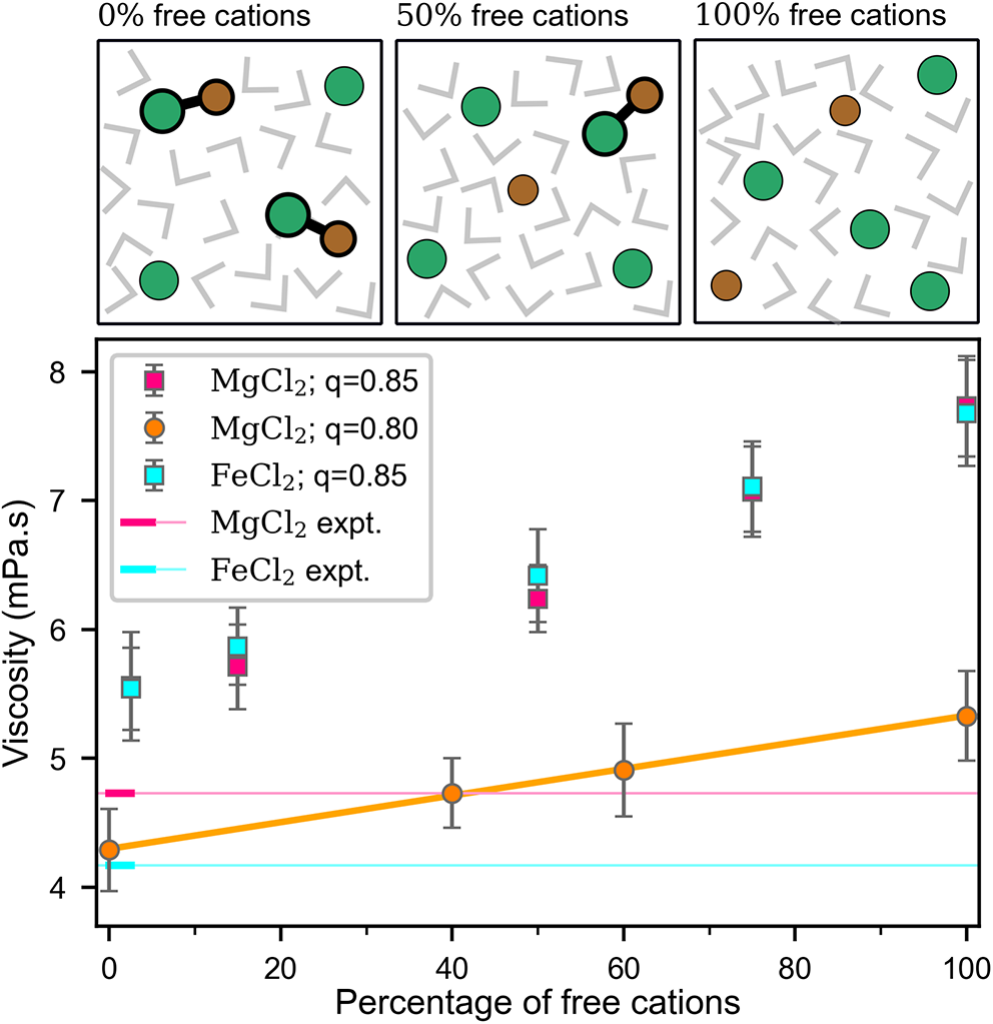
Experimentally measured viscosity of a 4 m solution of MgCl_2_ ^36^ and FeCl_2_ depicted as magenta and cyan horizontal lines, respectively, as well as the calculated viscosity (squares) of solutions containing a varying fraction of monomer complexes and free ions, shown as a function of the percentage of free cations, α. The orange circles depict calculated viscosity of MgCl_2_ using the Madrid-2019 force-field with a charge of 0.80 *e*. Insets on top illustrate solutions with no free cations, 50% free cations and only free cations.

The explanation of the decrease of viscosity with increase in complexes is intuitive: water molecules in the vicinity of ions diffuse more slowly than water molecules in the bulk.^62^ Additionally, the net charge of a monomer is half of that of a free cation, thereby exerting a weaker electric field on the surrounding water molecules. Thus, the presence of complexes (monomers, in this case) reduces the net amount of encumbered water molecules in contact with the ions, compared to solutions with free ions.

Consequently, the diffusion of water increases with a rise in the number of complexes, as shown in Table 4. The observation that the viscosity and the diffusion coefficient of water are anticorrelated in electrolyte solutions was discussed and shown by McCall and Douglass^63^ more than 60 years ago. This suggests that experimental measurements of *D*_H_2_O_ could also serve as an indirect measure of complex formation in these systems.

However, even after introducing monomers, quantitative agreement with experiments is not achieved. Figure 5 shows that even if we assume 100% population of monomers and no free cations, neither the viscosity of MgCl_2_ nor that of FeCl_2_ is reproduced. Of course, one should not disregard the presence of other complexes in the system, for instance those formed by the FeCl_2_ species (or the dimer species, in our terminology, see Figure 4).

Since dimers presumably hinder even less water molecules than monomers, we surmise that the presence of dimers should reduce the viscosity further. At 4 m with α″ = 100 (all the cations form dimers), the viscosity of the system (either MgCl_2_ or FeCl_2_) is calculated to be 4.8(0.2) mPa·s, which is lower than that of the system wherein all complexes form monomers [≈5.5 mPa·s]. However, this reduction is still not low enough to reach the experimental viscosity of FeCl_2_, which is 4.17 mPa·s (as shown in Table 2).

We also analyze the results of simulations with the 0.8 Madrid-2019 model, which tends to improve the description of transport properties, compared to the 0.85 model.^64,65^ Using this model, it is possible to reproduce the experimental viscosity of both MgCl_2_ and FeCl_2_, by assuming 40% of free cations for MgCl_2_ and 0% for FeCl_2_ (i.e., all cations participate in monomers), as shown in Figure 5 (Table S7 in the SI presents simulation results with 100% dimers). Our results indicate a 35–40% greater degree of complex formation in FeCl_2_ compared to MgCl_2_. Therefore, we surmise that the association for FeCl_2_ is significantly more important than for MgCl_2_, a conclusion supported by recent experiments.^18,32^

## 4 Discussion

### 4.1 Complex Populations: the Thermodynamic Formalism

Although experiments of FeCl_2_ solutions unanimously report the existence of complexes at high concentration, there is little consensus on the nature and type of dominant complexes formed.^11,17,18^ We speculate that determining their precise equilibrium distribution can be quite difficult from experiments, and, to the best of our knowledge, quantitative complex distributions have not been reported so far.

Another conceptually attractive route to estimating complex populations could be to obtain equilibrium constants. We define the equilibrium constant *K*_1_ for the reaction M^2+^ + Cl^–^ ⇌ MCl^+^ as

4 $$K_{1}=\frac{a_{\mathrm{MC}l^{+}}}{a_{M^{2+}}a_{Cl^{-}}}=\frac{\left[\mathrm{MC}l^{+}\right]}{[M^{2+}]\left[Cl^{-}\right]}\frac{\gamma_{\mathrm{MC}l^{+}}}{\gamma_{M^{2+}}\gamma_{Cl^{-}}}$$

where *M* refers to the metals considered here (Fe or Mg), *a*_i_ is the activity of component *i*, and γ_i_ is its activity coefficient, and [*i*] is the concentration of component *i*.

However, there is a wide disparity in the value of the estimated equilibrium constant *K*_1_. Table 5 presents log *K*_1_ from the literature, which varies from 0.74 to −0.89 for FeCl_2_, corresponding to a difference of almost 2 orders of magnitude. The value of log *K*_1_ of FeCl_2_ recommended by NIST is −0.2, which lies squarely in the middle of the range shown in Table 5. The same is true for MgCl_2_, although it has been studied much less than FeCl_2_,^13,23^ and the general consensus is that MgCl_2_ has low propensity to form complexes.^26,53,54^

**Table 5:** Equilibrium Constants log­(*K*_1_) for Monomer Formation of the Reaction M^2+^ + Cl^–^ ⇄ MCl^+^, where M Refers to the Metal

**FeCl**_**2**_	
Martell^12^	0.36
Wells and Salam^10^	0.74
Arnórsson et al.^13^	–0.40
Ruaya^14^	–0.50
Heinrich and Seward^15^	–0.16
Palmer and Hyde^16^	–0.125
Zhao and Pan^17^	–0.366
Böhm et al.^11^	–0.89
NIST	–0.2
**MgCl**_**2**_	
Ruaya^14^	–0.13
NIST	0.6

However, quantifying complex populations involves another contentious issue: activities (for instance, *a*_MCl^+^_, *a*_M^2+^_, *a*_Cl^–^_), and not concentrations, need to be estimated for real solutions. Individual activity coefficients cannot be directly calculated from experiments. Activity models, such as Specific Ion Theory (SIT)^19–21^ or the Davies equation^22^ are often used to estimate complex populations, given equilibrium constant values. Chemical equilibrium models, such as Visual MINTEQ (version 4.0),^23^ can estimate percentage distributions of free cations, using such activity models. However, even when starting from the same equilibrium constant (for instance, those recommended by NIST), different activity models yield widely diverging complex populations, as shown in Table 6.

**Table 6:** Percentage of Free Cations, α, in 4 m Aqueous Solutions at Room Temperature and Pressure, Obtained from Visual MINTEQ^23^ using Different Activity Models and Association Constants from NIST^a^

	activity model		
system	SIT	Davies	γ = 1
MgCl_2_	8%	0.5%	6%
FeCl_2_	29%	3%	24%

a The column labeled γ = 1 refers to results obtained from eq 4 using the approximation that all activity coefficients are unity. More association is predicted for MgCl_2_ than for FeCl_2_.

As can be expected, calculations using such chemical equilibrium models are sensitive to the quality of the activity model and the ion association constants. We surmise that, at this point, they are not reliable enough to unambiguously determine the concentration of complexes.

### 4.2 Toy Model Exploiting the Relationship between Complex Populations and Viscosity

In Section 3.3.2, we presented a model that could reproduce the experimental viscosities of FeCl_2_ and MgCl_2_ simultaneously, contingent on a particular (variable) complex population used to fit to the viscosity (solid orange line in Figure 5).

Simulation results notwithstanding, we cannot claim to quantitatively determine the equilibrium concentration and the type of complexes that actually exist experimentally in FeCl_2_ and MgCl_2_. However, we will try to develop a toy model that, although approximate, can at least qualitatively describe the high concentration viscosity trends of FeCl_2_ and MgCl_2_.

We introduce a second equilibrium constant, *K*_2_, defined as the equilibrium constant for the reaction MCl^+^ + Cl^–^ ⇌ MCl_2_^0^ according to

5 $$K_{2}=\frac{a_{\mathrm{MC}l_{2}^{0}}}{a_{\mathrm{MC}l^{+}}a_{Cl^{-}}}=\frac{\left[\mathrm{MC}l_{2}^{0}\right]}{[\mathrm{MC}l^{+}]\left[Cl^{-}\right]}\frac{\gamma_{\mathrm{MC}l_{2}^{0}}}{\gamma_{\mathrm{MC}l^{+}}\gamma_{Cl^{-}}}$$

where symbols have the same meaning as in eq 4.

In addition to relations for the equilibrium constants, we need a model to describe how the viscosity varies as the population of complexes changes. We propose a simple and heuristic approach, and suggest the following equation to estimate the viscosity at 4 m for MgCl_2_ and FeCl_2_

6 $$\eta \left(4m\right)=\eta^{0}-\delta^{\prime }\alpha^{\prime }-\delta^{\prime \prime }\alpha^{\prime \prime }$$

where η^0^ is the viscosity (in mPa·s) of a solution with 100% free cations.

The basis for this formula is the observation that the viscosity changes linearly with the number of monomers, in the absence of dimers, and also that the viscosity changes linearly (albeit with a different slope) with the number of dimers, in the absence of monomers. The value of the slopes, can be obtained from the simulations of this work. We obtain values of δ_*q* = 0.85 *e*_^′^ = 0.020 mPa·s and δ_*q* = 0.80 *e*_^′^ = 0.010 mPa·s for the *q* = 0.85 *e* and *q* = 0.80 *e* models, respectively, from the slopes in Figure 5. For δ″, we obtain δ_*q* = 0.85 *e*_^″^ = 0.029 mPa·s and δ_*q* = 0.8 *e*_^″^ = 0.014 mPa·s (derived in Section 11 in the SI).

Second, we need the values of the association constants *K*_1_ and *K*_2_. We shall use the NIST value for *K*_1_ of FeCl_2_ [i.e., log­(*K*_1_) = −0.20]. However, obtaining a value for *K*_2_ is more cumbersome. As shown in Section 11 of the SI, log *K*_2_ is often around 0.8 units smaller than log *K*_1_ (see also Table 6.1 in Burgess^57^). Therefore, we adopt the values of log *K*_2_ for FeCl_2_ and MgCl_2_ listed in Table 7. Note that we imposed a smaller value of *K*_1_ for MgCl_2_, compared to that of FeCl_2_. This was motivated by the observation that MgCl_2_ is an archetypal strong 2:1 electrolyte that exhibits little complexation in concentrated solutions.^54^

**Table 7:** Values of Equilibrium Constants^a^ used for a Qualitative Calculation of the Viscosity of FeCl_2_ and MgCl_2_ at 4 m, Estimated Complex Distributions (Exemplified by α, α′, α″), and the Estimated Viscosity, *η*, Calculated from eq 6, using *η* ^0^ = 5.13 mPa·s (the Value of the Viscosity Obtained with the 0.8 Charge Model) for Both Solutions

system	log *K*_1_	log *K*_2_	α	α′	α″	η (mPa·s)
FeCl_2_	–0.20	–1.0	22	56	22	4.46
MgCl_2_	–1.1	–1.9	64	33	3	4.96

a Obtained from NIST, Lange’s Handbook^66^ or estimated using reasonable guesses; see also Section 11 of the SI.

Typically, the values of *K* are determined from the concentrations at infinite dilution, where the activity coefficients tend toward one. However, at finite concentrations, individual activity coefficients should be estimated, which is usually done using theoretical models, such as SIT^19–21^ (see also Section 4.1) or via simulations,^67^ using certain approximations. Note that what is needed is not necessarily each individual activity coefficient, but the right-hand side of eqs 4 or 5.

It is true that this term is probably not equal to one. Whatever the true value of this term is, one could consider this term to be effectively incorporated into the value of *K*_1_ and *K*_2_, thus defining an effective equilibrium constant *K*_1_^*^ or *K*_2_^*^. These are not true thermodynamic equilibrium constants but they are, instead, effective constants that are used with concentrations, and not with activities (they are usually denoted in the literature as stoichiometric equilibrium constants,^68^ and in contrast to the true equilibrium constants that depend only on temperature, these constants also depend on the media in which the reaction takes place). In this illustrative calculation, we assume that all values of γ are unity and that Table 7 reports the true equilibrium constant, or that, equivalently, Table 7 reports the values of stoichiometric equilibrium constants *K*_1_^*^ and *K*_2_^*^ (with which one would use the concentrations and not activities). In any case, our approach to the problem is meant to be qualitative, rather than quantitative. Moreover, this assumption yielded free cation populations which are in good agreement with the SIT model, when only monomers were considered, as shown in Table 6.

Thus, with the values of the equilibrium constants of Table 7 and using eq 6 to predict the viscosity and the results of the force field with the scaled charge 0.8, we obtain a viscosity of 4.46 mPa·s for FeCl_2_ (compared to 4.17 mPa·s from our experiments; see Table 2) and 4.96 mPa·s for MgCl_2_ (compared to the experimental value of 4.73 mPa·s; see also Section 2 in the SI), respectively. Therefore, agreement with experiments is very good, within 5%, which is also consistent with our simulation results. The populations of free ions, monomers and dimers are presented in Table 7. This simple toy model is consistent with the existence of monomers and dimers in FeCl_2_ solutions, as shown in recent experiments.^17,18^

However, we emphasize that we are not claiming that the complex distributions obtained from this calculation correspond to those found in real solutions of FeCl_2_ and MgCl_2_. We are merely showing how a certain population of complexes would be compatible with the experimental values of the viscosities. Note that there could be several complex distributions that are consistent with the experimental values. The solid orange line in Figure 5 (which assumes the absence of dimers) is also able to describe the viscosities of both solutions. However, in every case examined in this work, there is more association in FeCl_2_ than in MgCl_2_, regardless of the exact proportions of complexes estimated in each solution. In the future, properties such as osmotic coefficients,^46^ freezing point depression,^69^ or electrical conductivities^70^ could be useful for evaluating different speciation possibilities, since we expect them to also be sensitive to the presence of complexes.

### 4.3 Limitations of Empirical Force-Fields

Most of the ions described up to this point by the Madrid-2019 force-field have an electronic configuration close to that of noble gases (halogen, alkaline, alkaline-earth), and for those the model has been relatively successful. Modeling Fe^2+^ with this force-field is the first foray into transition metals which has revealed various challenges and nuances tied to complex formation. An interesting future challenge will be a study of solvated transition metal ions with larger charge, such as Fe^3+^. Recent simulations have shown abrupt switching between two different solvation shell structures, with lifetimes of the order of nanoseconds.^71^

However, even with an empirical force-field, it is possible to increase the population of FeCl^+^ monomers with respect to MgCl^+^. This could be accomplished by having different LJ parameters for the Fe–Cl and Mg–Cl interactions. For instance, decreasing the value of σ (the size parameter in the LJ potential), in the first case, with respect to the second, would increase the strength of the Mg–Cl interaction and would consequently increase the population of monomers. This approach was used by Dubouè-Dijon et al.^26^ to increase the number of CIPs in ZnCl_2_. Although this methodology is interesting, we believe that it is more fruitful to recognize the limits of empirical force fields to quantitatively describe the energy between a transition metal with several 3 *d* valence electrons and a ligand, and to acknowledge that only an electronic structure calculation can describe this interaction in a quantitative way. Since the required size of a simulated system is large and the computational effort of electronic structure calculations scales rapidly with the number of electrons, a practical approach could involve a hybrid simulation method, such as QM/MM (quantum mechanics/molecular mechanics).^72^

## 5 Conclusions

The literature contains numerous conflicting reports on complex speciation and the extent of complexation in concentrated FeCl_2_ and MgCl_2_ solutions. In general, using experiments, theory or simulations to estimate equilibrium complex populations in concentrated solutions is a nontrivial task. However, knowledge of speciation is crucial to understand the behavior of solutions.

Modeling a transition metal, such as Fe^2+^, with an empirical force-field is complicated by electronic structure effects (for instance, 3d^6^ configuration). In light of this, the extension of the Madrid-2019 force-field to Fe^2+^ may be deceptively simple, but is a research outcome in its own right.

We have introduced a methodology to incorporate complexes in our simulations which is similar, in spirit, to the seeding method for nucleation,^73–75^ wherein prefabricated critical clusters are introduced. Both methods are characterized by two different time scales: a significantly large one (corresponding to the attainment of the equilibrium complex population in concentrated solutions, in this case, or to the emergence of a critical nucleus in nucleation), and another relatively short time scale of interest (corresponding to transport property calculations as in this work, or to the evolution of a critical cluster in nucleation). Inserting complex populations in simulations of concentrated solutions *a priori* enables us to bypass the computationally prohibitive time required to reach these equilibrium populations. Note that one could obtain information about plausible complex populations from various sources, such as experiments, first-principles, and thermodynamics, and subsequently perform simulations using any standard force-field.

We have presented an argument for a strong link between complex distributions and the viscosity, using computer simulations (wherein input complex populations were varied) and with new experimental data for FeCl_2_ at high concentration. Although the methods described in this work cannot quantitatively estimate complex populations, our results provide qualitative information about speciation and show that FeCl_2_ has a higher propensity for complexation than MgCl_2_. This could be particularly valuable to obtain clarity about complex speciation when experimental data is elusive or inconclusive.

This idea is not as novel as it may first seem. Weingartner et al.^9^ previously reported experimental viscosity measurements for MgCl_2_ and ZnCl_2_, observing that the two systems exhibit very similar viscosities at low concentrations but diverge significantly at higher concentrations. The authors speculated that the formation of complexes caused the lower viscosity of ZnCl_2_. While one cannot change the number of complexes at will in experiments, this can be easily achieved in simulations. In this work, we have demonstrated how complexes can affect the viscosity of solutions at high concentrations, thus confirming the intuition of Weingartner et al.^9^ Nevertheless, further experimental work is required to quantitatively clarify the amount of complexes in concentrated solutions, and NDIS (Neutron Diffraction with Isotopic Substitution^76–78^ ), using chloride isotopes in “null” water (a mixture of H_2_O and D_2_O),^46^ could be particularly informative, especially since the chloride-oxygen chloride-cation peaks would not overlap (expected to be around 3.14 and 2.33 Å, respectively). We hope that future research will be continued in this direction, which was first envisaged by Weingartner et al.^9^ more than 40 years ago in this journal.

## Supplementary Material



## Notes

A workflow for creating configurations, running LAMMPS simulations, and calculating the viscosity can be found at https://github.com/amritagos/electrolyte_workflow. The Supporting Information contains a description of the simulation methodology for molecular dynamics (MD) simulations, details about the experimental viscosity of MgCl_2_ used in this work, an expanded discussion of the justifications for using the force-field parameters of Mg^2+^ for Fe^2+^, rationale for the cation-chloride distances chosen to create complexes in simulations (by freezing the distance) and further justifications for this approach, an analysis of solvation shell structures showing that freezing cation-chloride distances does not change the solvation shell, theoretically expected viscosity trends of MgCl_2_ and FeCl_2_ and details about approximating electrolyte solutions as LJ fluids in order to estimate expected viscosity, predictions of complex speciation from Visual MINTEQ, densities calculated from simulations with and without complexes, a discussion of the slight overestimation of the viscosity by the Madrid-2019 force-field, the effects of complex populations on transport properties, additional details about a back-of-the-envelope calculation for estimating viscosity from eq 6, and details of how Jones-Doles *B* coefficients were estimated using the force-field (PDF)

## ⊥

Departamento de Química Física, Facultad de Ciencias Químicas, Universidad Complutense de Madrid, 28040 Madrid, Spain
