# Supplementary material for: Viscosity as a Smoking Gun for Complex Formation in Solution: Fe2+ and Mg2+ Chlorides as Examples
Source: J Phys Chem B. 2026 Mar 16;130(12):3436–46. doi: 10.1021/acs.jpcb.6c01329 (PMC13034410; doi:10.1021/acs.jpcb.6c01329)
Supplement: Supplementary file 1 [file jp6c01329_si_001.pdf]

Supporting Information: Viscosity as a Smoking Gun for Complex Formation in Solution: Fe^{2+} and Mg^{2+} Chlorides as Examples

Amrita Goswami,[†] Samuel Blazquez,[‡] Lucía Fernández-Sedano Vázquez,[‡] Eva González Noya,[¶] Hannes Jónsson,[†] Jacobo Troncoso,[§] and Carlos Vega*,[‡]

†*Science Institute and Faculty of Physical Sciences, University of Iceland, VR-III, 107 Reykjavík, Iceland*

‡*Departamento de Química Física, Facultad de Ciencias Químicas, Universidad Complutense de Madrid, 28040 Madrid, Spain.*

¶ *Instituto de Química Física Blas Cabrera, CSIC, C/Serrano 119, 28006 Madrid, Spain.*

§*Departamento de Física Aplicada, Universidade de Vigo, Escola de Enxeñaría Aeronaútica e do Espazo, E 32004, Ourense, Spain*

E-mail: cvega@ucm.es

Contents

1 Simulation methods **S2**

1.1 Simulation details S3

1.1.1 GROMACS S4

1.1.2 LAMMPS S5

1.2 Configuration generation with complexes and energy minimization S5
1.3 Viscosity calculations from simulations S6
2 Experimental viscosities of $MgCl_2$ S7
3 Justification for the Fe^{2+} force-field parameters S7
4 Justification for chosen cation-chloride distances and for rigid bonds S9
5 Validation of simulation protocol with rigid cation-chloride bonds S10
6 Theoretically expected viscosity trends of $FeCl_2$ and $MgCl_2$ S11
7 Predictions of speciation from chemical equilibrium models S13
8 Densities from simulations S14
8.1 Simulations without complexes S14
8.2 Simulations incorporating complexes S15
9 Overestimation of viscosities by Madrid-2019 S15
10 Speciation effects on transport properties S16
11 Back-of-the-envelope calculation for viscosities S18
12 Evaluation of the Jones-Dole coefficient of a force-field S20
References S21

1 Simulation methods

In this work, we have extended the Madrid-2019 force-field[S1] to the Ferrous ion, Fe^{2+}, using the same parameters as for Mg^{2+}. The Madrid-2019 force-field combines the TIP4P/2005 model of

water[S2] with scaled charges for the ions. The proposed force-field parameters are collected in Table 1 in the main text. Parameters for Mg^{2+}, Cl^- can be found in our previous work[S1] and those of perchlorate from our recent work.[S3] Note that the canonical Madrid-2019 force-field[S1] uses a scaled charge of 0.85 e. It is well-known that the Madrid-2019 force-field tends to overestimate the viscosities of electrolytes in water (even for NaCl, KCl where contact ion pairs, but no complexes, are formed), as described further in Sec 9. It has been proposed that lowering the charge to 0.8 reduces many of the discrepancies with experiments (and with 0.75 it is even possible to reproduce the experimental results).[S4] For this reason, we have also performed analyses using a scaled charge of 0.8. The change of the charge of the ions requires some adjustment of the Lennard-Jones (LJ) parameters (simply replacing the charge and keeping the LJ parameters optimized for the $q = 0.85$ e model is not a good option[S4]). For the model with $q = 0.8$ e, the value of σ for the $Cl-O_w$ was taken from our previous work Ref.[S4] and the value of LJ σ for the $Fe^{2+}-O_w$ or $Mg^{2+}-O_w$ was optimized in this work, obtaining $\sigma = 1.85$ Å, as shown in Table 1 in the main text.

Regarding $FeCl_2$ simulations, it is important to consider the possibility of Fe^{2+} undergoing a certain amount of hydrolysis in water. However, in this work, for the highest concentration (4 m), the pH is about 5. Therefore, the concentration of H_3O^+ and $Fe(OH)^+$ is quite small (of the order of about 10^{-5} m) and can be neglected when performing simulations to describe this system.

We have used the molality scale, m, for concentrations (mol of salt per kg of water) as is typically done in experimental studies, since the concentration then does not depend on the volume of the system. Furthermore, in simulations we typically used 555 molecules of water to determine the densities of the aqueous ionic solutions (and multiples of the same for viscosity simulations). This is very convenient, since then 1 m or 2 m solutions are obtained with 10 or 20 molecules of salt.

1.1 Simulation details

Simulations were performed using the GROMACS (version 2025.2)[S5] and LAMMPS (29 Aug 2024, Update 1)[S6] packages. Specific simulation details for GROMACS and LAMMPS are pro-

vided in Sec. 1.1.1 and Sec. 1.1.2, respectively.

Densities were computed from NpT simulations, with 555 water molecules, run for 50 ns each. Prior to production runs, the simulations were equilibrated for 10 ns.

For the viscosity calculations, each independent configuration contained 4440 water molecules, within a box size corresponding to the averaged density for each concentration. Note that independent configurations were created using the methodology described in Sec. 1.2. Viscosities were computed from 8 – 15 independent simulation runs in the NVT ensemble, using both LAMMPS and GROMACS, which were subsequently averaged (over a total of 16 – 30 independent simulations). Each independent simulation was run for 40-50 ns (following a 4 ns simulation of simulated annealing and subsequently a 20 ns period of equilibration).

The diffusion coefficient was calculated from unwrapped coordinate trajectories generated during the viscosity calculations in the NVT ensemble. The Yeh-Hummer correction[S7] was applied to the diffusion coefficients to account for finite-size effects.

A workflow for creating configurations, running LAMMPS simulations and calculating the viscosity can be found at `https://github.com/amritagos/electrolyte_workflow`. Snapshots and visuals were created using `solvis` (`https://github.com/amritagos/solvis`).

1.1.1 GROMACS

In GROMACS, simulations were performed in the NpT ensemble with a Nose-Hoover thermostat[S8] and the barostat from Parrinello-Rahman.[S9,S10] The time step was of 2 fs. Ewald sums (PME) were used for the long-range electrostatic interactions in GROMACS. A long-range correction to the LJ part of the potential was added. The potential was truncated at 10 Å. The shape of the water molecules was constrained using the LINCS algorithm.[S11] In the case of complexes, the cation-anion distance was also frozen using LINCS (see also Sec.4 and Sec.5).

1.1.2 LAMMPS

Analogous simulations in LAMMPS were performed using the Nose-Hoover thermostat[S8] and Nose-Hoover barostat,[S12] with a thermostat and barostat coupling constant of 200 fs and 5 ps, respectively. Likewise, the timestep used was 2 fs. Long-range electrostatics were computed using particle-particle particle-mesh (PPPM).[S13] Similar to the protocol in GROMACS, long-range corrections to the LJ terms were added, and a cutoff of 10 Å was used. The SHAKE algorithm[S14] was used to constrain the shape of water molecules and to constrain the cation-anion distances in complexes (see also Sec.4 and Sec.5).

1.2 Configuration generation with complexes and energy minimization

As part of our new simulation strategy of studying transport properties at a static predetermined complex population (see Sec.4 and Sec.5 for justification and validation), we perform simulations in which we artificially maintain the desired complex population. This is accomplished by constraining the cation-anion distance in simulations of $FeCl_2$ and $MgCl_2$. Concerning configuration generation, usually it is trivial to create initial configurations using tools such as PACKMOL[S15] or Moltemplate.[S16] However, especially for concentrated systems, and in the situations where we insert complexes, configuration creation can be a slightly more involved process. In this work, a *monomer* refers to a complex formed by one cation, coordinated with one Cl^- anion and some additional molecules of water in the first solvation shell, while a *dimer* refers to a complex wherein there are two Cl^- anions in the trans position of the octahedron, and some additional coordinating water molecules (see Fig.2 in the main text). In the case of a "monomer" or "dimer" complex, a unit consisting of a bonded metal ion and chloride, or a "triatomic" unit consisting of two chlorides and one metal ion was created. Each species was randomly distributed in the simulation box, keeping a tolerance of 3.0 Å between complexes. A `Python` program was written for this purpose which uses PACKMOL, Moltemplate and Atomic Simulation Environment (ASE),[S17] under the hood; see `https://github.com/amritagos/confi`. Certain typical file formats in LAMMPS and GROMACS can also be converted using `confi`.

Configurations were created in the manner described above in order to facilitate subsequent energy minimization. Energy minimization with the aforementioned complexes was performed by keeping the complexes frozen and only allowing the water molecules to move. However, in the case of free cations and anions, all atoms were allowed to move. The energy was minimized in two stages– first with 1000 steps of steepest descent, followed by a maximum of 10000 steps of the conjugate gradient algorithm, with a step size of 0.001 fs.

1.3 Viscosity calculations from simulations

Using the Green-Kubo formalism, the viscosity is obtained from the expression:

$$\eta = \frac{V}{k_B T} \int_0^{\infty} \langle P_{\lambda\beta}(\tau)\, P_{\lambda\beta}(0) \rangle \, d\tau, \tag{S1}$$

with k_B the Boltzmann constant, V is the volume, T is the temperature and $P_{\lambda\beta}$ are the non diagonal components of the pressure tensor (i.e., $\lambda \neq \beta$) which are given as:

$$\begin{aligned} P_{\lambda\beta}(\tau) =& \frac{1}{V}\sum_{i=1}^{N} m_i v_{\lambda,i}(\tau) v_{\beta,i}(\tau) \\ &+ \frac{1}{V}\sum_{i=1}^{N} r_{\lambda,i}(\tau) f_{\beta,i}(\tau) \\ =& P^K_{\lambda\beta} + P^U_{\lambda\beta}, \end{aligned} \tag{S2}$$

where $v_{\lambda,i}$, $r_{\lambda,i}$ and $f_{\lambda,i}$ denote the λ component of the velocity, position and force acting on particle i respectively. The first term of Eq. (S2) is a kinetic term that depends on the masses of the particles of the system, and the second term is a virial term that only depends on positions and forces, but not on masses. The sum in Eq. (S2) is over the N particles of the system (water and ions, in our case). The three non diagonal components and the terms $\frac{1}{2}\,(P_{xx} - P_{yy})$ and $\frac{1}{2}\,(P_{yy} - P_{zz})$ were used to get the correlation function of the pressure tensor as in the work of Gonzalez and Abascal.[S18] The pressure tensor can be divided into a kinetic contribution K, and a potential energy contribution U as shown in Eq. (S2). Therefore when using the Green-Kubo formula one has a KK

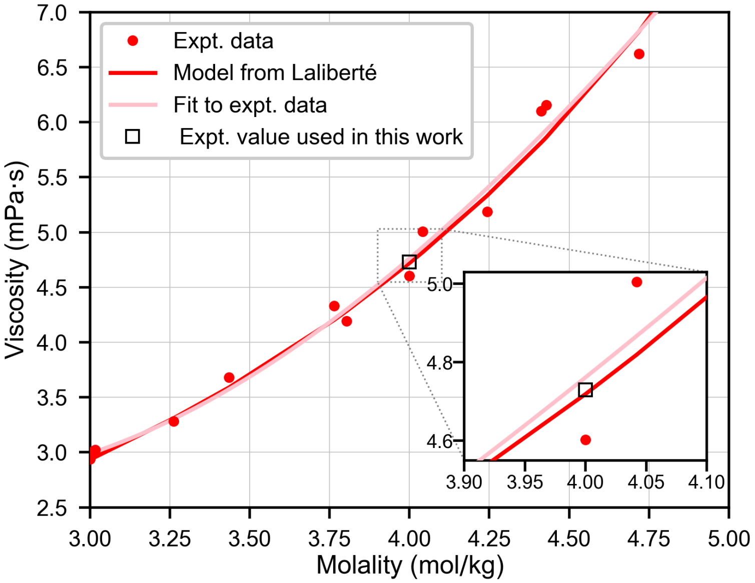

Figure S1: Viscosities of $MgCl_2$ from experiments[S19] (filled red circles), model fitted to experimental data points[S19] (solid red line), along with a fit to experimental data points in the interval 3 m to 5 m (solid pink line). The experimental value of the viscosity used in this work at 4 m is denoted by an open square. The inset shows a zoomed-in view close to 4 m.

contribution, a KU contribution and a UU contribution.

2 Experimental viscosities of $MgCl_2$

As shown in Fig. S1, individual experimental measurements (red circles) are scarce and rather scattered. Therefore, Laliberte[S19] has proposed a model that fits to the existing experimental data (solid red line in Fig. S1). In this work, we have performed a detailed study at 4 m. Therefore, we have also fitted to the experimental data in the range 3-5 m. Using the model from Laliberte[S19] and our own fit, we settle on a viscosity of 4.73 mPa·s for $MgCl_2$ at 4 m. Subsequently, we have used this value in this work as the experimental value of $MgCl_2$.

3 Justification for the Fe^{2+} force-field parameters

Fig. S2(a) presents the densities of $FeCl_2$ and $MgCl_2$ obtained from experiments, including the experimental results of this work (see also Table 2 of the main text). As can be gleaned from

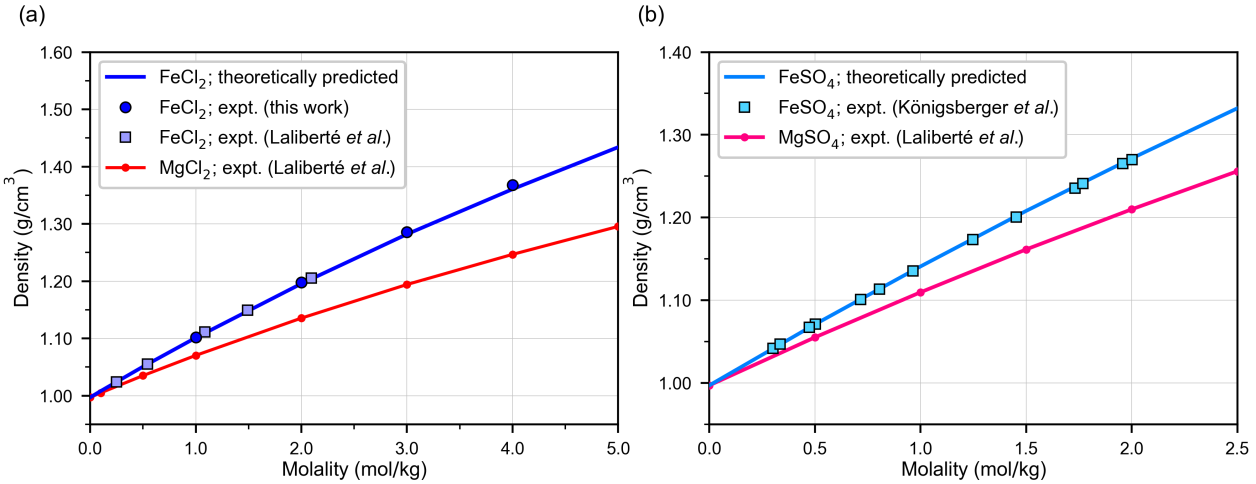

Figure S2: (a) Experimental densities of $FeCl_2$ (light-blue symbols and blue symbols for results from Laliberté and Cooper[S20] and results from this work, respectively), as well as experimental densities of $MgCl_2$ (red line). Assuming equal volumes for the $FeCl_2$ and $MgCl_2$ solutions, we obtain the solid blue line as the predicted density of $FeCl_2$. (b) Densities of $FeSO_4$ and $MgSO_4$ from experiments, and those scaled by assuming equal volumes. Densities of $FeSO_4$ were taken from Königsberger et al.[S21], and those of $MgSO_4$ from Laliberté and Cooper[S20].

Fig. S2(a), the mass densities of $FeCl_2$ and $MgCl_2$ are clearly different.

Using the approximation of equal number densities (Eq. 1 in the main text), we can theoretically estimate the mass density of $FeCl_2$, using the experimentally calculated mass densities of $MgCl_2$. Fig. S2(a) shows that the theoretically predicted mass densities of $FeCl_2$ (assuming that $MgCl_2$ and $FeCl_2$ have the same number density) agree well with the densities from experiments. We show that a similar approximation can be made for $FeSO_4$ and $MgSO_4$ solutions in Fig. S2(b).

This is a promising sign that the same force-field parameters as Mg^{2+} could be used for Fe^{2+}.

Table S1: Metal – O_w distances, d_{M-O_w} (in Å), and hydration free energies (in kcal/mol).

	d_{M-O_w}	HFE
$Mg^{2+} - O_w$	2.11 ± 0.01^b	-437.4^c
$Fe^{2+} - O_w$	2.095^a<br>2.09 ± 0.04^b	-439.8^c

* a) From Uroš Luin et al.[S22] b) Collected in Ohtaki and Radnai[S23], Marcus[S24] c) From Marcus[S25]

We believe that this is further justified by the fact that the experimentally measured cation-

oxygen distance[S26] is similar for Mg^{2+} and Fe^{2+}, as shown in Table S1. Table S1 also presents the hydration free energies, which are almost identical for both ions. Therefore, we surmise that the cation-water interactions for both cations are similar, both in terms of the distance and magnitude of the interaction energy. Following on this premise, we conclude that a suitable force-field for Mg^{2+} should work reasonably well for Fe^{2+}.

Note that when the same force-field is used to describe Mg^{2+} and Fe^{2+}, despite having different masses, classical simulations should necessarily yield the same thermodynamic properties such as the hydration free energy, radial distribution functions, surface tension, activity coefficients and freezing point depression. This is a consequence of using classical statistical thermodynamics. However, transport properties are affected by the mass and are, therefore, expected to be different.

4 Justification for chosen cation-chloride distances and for rigid bonds

Table S2: Bond length distances, d_{M-Cl}, (in Å) in the metal ion chloride (M – Cl) complexes.

System	d_{M-Cl}
Mg^{2+}– Cl	2.30[a], 2.55[b]
Fe^{2+}– Cl	2.33[c]
Fe^{3+}– Cl	2.30[d]

[*] a) From a simulation study by Zapałowski and Bartczak[S27] b) From an experimental study of $MgCl_2(H_2O)_4$ by Schmidt et al.[S28] c) From Uroš Luin et al.[S22] d) From Lind[S29]

It has been shown that when the metal–water oxygen distance (M–O_w) is similar for two cations, their corresponding metal–chloride (M–Cl) distances are also comparable. For example, Kuppuraj et al.[S30] demonstrated this relationship for the divalent cations Co^{2+} and Fe^{2+}.

Metal–chloride distances for a range of divalent metal ions are reported in Refs.[S31,S32] and are summarized in Table S2. These data show that the M–Cl bond lengths for Fe^{2+} and Mg^{2+} fall

within a narrow range.

On this basis, we adopt the same metal–chloride bond distance for both the Fe–Cl and Mg–Cl complexes, setting M–Cl = 2.33 Å. Note that, in our new simulation approach, cation-anion distances are constrained to the same value using either the SHAKE[S14] or LINCS[S11] algorithms.

We justify this new approach of freezing ion pairs in the following. The lifetime of the cation-anion pair (complexes) in $FeCl_2$ and $MgCl_2$ is of the order of microseconds.[S33] Note that this means that a chloride ion cannot leave the Fe^{2+} or Mg^{2+} cation within our simulation time scales. However, insofar as the viscosity is concerned, a lifetime greater than $\approx$ 100 ps could be effectively considered "intransient" or "stable". This is because the Green-Kubo integral reaches a plateau, and that indicates that the integrand, which gives the correlation function of the non-diagonal pressure tensor components, goes to zero, as can be seen from the inset of Fig. 1 in the main text. Thus, if the residence time of a cation and an anion is less than 100 ps (which is the case for aqueous NaCl, KCl or RbCl), then it is a labile contact ion pair, and it is more useful to consider it as a fluctuation within the configurational space.

As touched upon previously, the stability of long-lived complexes formed in $FeCl_2$ and $MgCl_2$ implies that reaching the equilibrium chemical composition can take of the order of microseconds to seconds (especially if the empirical force-field, or even an *ab-initio* simulation, mimics the experimental relaxation times, in a realistic way, which is desirable). When using the Madrid-2019 force-field, the formation of complexes is not observed even after 200 ns runs (i.e., the cation and anion were not in contact, in both the initial and in the final configurations).

5 Validation of simulation protocol with rigid cation-chloride bonds

To assess the validity of the aforementioned simulation strategy, we analyzed the structures of the solvation shell around free cations, monomers and dimers. The radial distribution functions between Mg^{2+} and O_w, for free cations, monomers and dimers, at a concentration of 4 m are shown

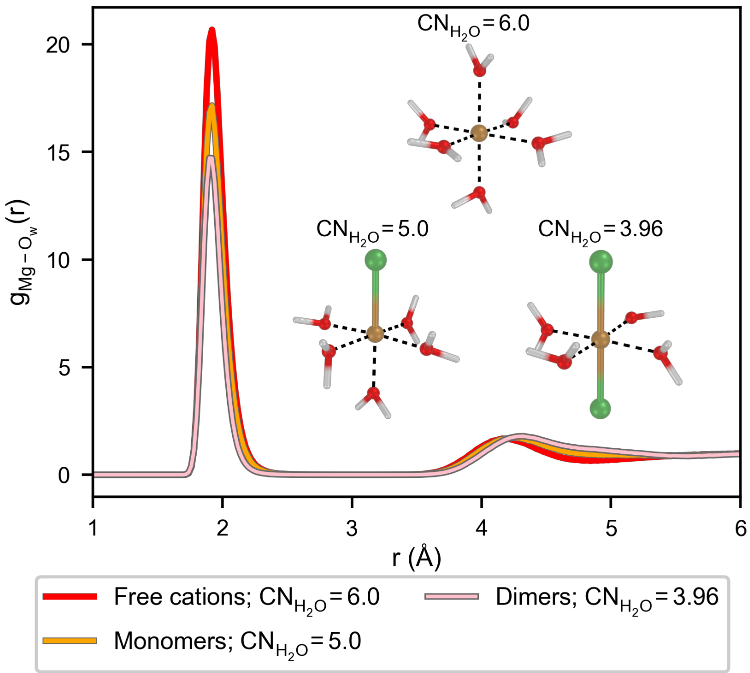

Figure S3: Radial distribution function between Mg^{2+} and the oxygen atoms in water obtained from simulations using free ions (red curve), the monomer complex (yellow curve), the dimer complex (pink curve), and the water coordination number (CN_{H_2O}) at 4 m. The total coordination number (including both the water molecules and chloride ions in the first solvation shell) is close to 6.0 in all cases. Insets depict typical solvation shells for free cations, monomers and dimers.

in Fig. S3. The expected solvation structure around both Mg^{2+} and Fe^{2+} is octahedral.[S23,S34] If this structure is retained by the monomers and dimers, the total coordination number should be close to 6.0. Since we obtain hydration numbers of 5.0 and 3.96 for monomers and dimers, respectively, as shown in Fig. S3, we can conclude that constraining the cation-chloride distance does not distort the solvation shell around the cation.

6 Theoretically expected viscosity trends of $FeCl_2$ and $MgCl_2$

From Fig.2 in the main text, it is evident that while the viscosities of $MgCl_2$ and $FeCl_2$ are similar at low concentration, upto 2 m, they diverge greatly at higher concentration. From experiments, the viscosity of $FeCl_2$ at 4 m is 4.17 mPa.s (Table 1 in the main text), compared to that of $MgCl_2$ at the same concentration, equal to 4.73 mPa.s (see also Sec. 2). In the main text, we conclude that

the formation of complexes (greater in $FeCl_2$ compared to $MgCl_2$) is responsible for the observed viscosity trends.

Here, we rule out other possible explanations of the trends. One could speculate that the difference in mass is responsible for the observed difference in viscosity, but there are several reasons to surmise that this is not actually the case. The mass of the two most abundant components in both solutions, water and chloride, is the same. The cation is the least abundant component, and the mass of Fe is only 2.5 times larger than that of Mg.

Furthermore, we approximate the $FeCl_2$ and $MgCl_2$ solutions as effective Lennard-Jones systems, for the sake of a qualitative argument that further illustrates our point. Meier and coworkers[S35] have shown that, in a Lennard-Jones (LJ) system, the KK and KU integrals are much smaller than the UU integral [see Eqs. (S1) and (S2)], which is the main contribution. For such an LJ system, the viscosity, in reduced units, is universal (i.e., it does not depend on the masses) and it is given in units of $\eta^* = \eta\sigma^2/\sqrt{m\varepsilon}$. In other words, for two systems with the same LJ parameters, the value of the viscosity is proportional to $\sqrt{m}$. The average mass of a particle (performing the average over molecules of water, cations and anions) in the 4 m $FeCl_2$ solution is 22.32 g/mol, whereas in the $MgCl_2$ solution it is 20.46 g/mol. Thus, one would expect the viscosity of the $FeCl_2$ solution to be about $\sqrt{22.32/20.46} = 1.05$ (i.e., 5% larger) than that of $MgCl_2$. However, in the experiments, the viscosity of $FeCl_2$ is 12% lower than that of $MgCl_2$, resulting in a deviation from the expected result of about 17%. Consequently, we conclude that the mass is not responsible for the observed differences in experimental viscosity at high concentrations. Moreover, we believe that the overestimation of viscosity obtained from our simulations for both $MgCl_2$ and $FeCl_2$ is not explained by a known tendency of the Madrid-2019 model to overestimate viscosity (see Sec. 9 for more details).

7 Predictions of speciation from chemical equilibrium models

A popular (and free) program is Visual MINTEQ,[S36] that enables the estimation of the population of complexes at 4 m for $FeCl_2$ and $MgCl_2$. This program uses the association constants (at infinite dilution) recommended by NIST, and obtains the equilibrium population after solving the equation of balance of mass, charge, iteratively, until all equilibrium constants and user-defined constraints (the concentration, pH, etc) are satisfied. In principle, this seems like a reasonably sound strategy, but these calculations can be sensitive to 1) the association constants, and 2) the activity model used, such as the Specific Ion Theory[S37–S39] (SIT), Davies equation[S40] , etc. As detailed in the main text, experiments differ widely in the estimation of association constants.

The program clearly predicts complex formation in both $MgCl_2$ and $FeCl_2$ solutions. We emphasize that the quantitative complex populations predicted depend strongly on the chosen activity model. However, independent of the activity model, Visual MINTEQ predicts higher association in $MgCl_2$ compared to $FeCl_2$, which goes against the expectation of less complex formation in $MgCl_2$.[S41–S43]

8 Densities from simulations

8.1 Simulations without complexes

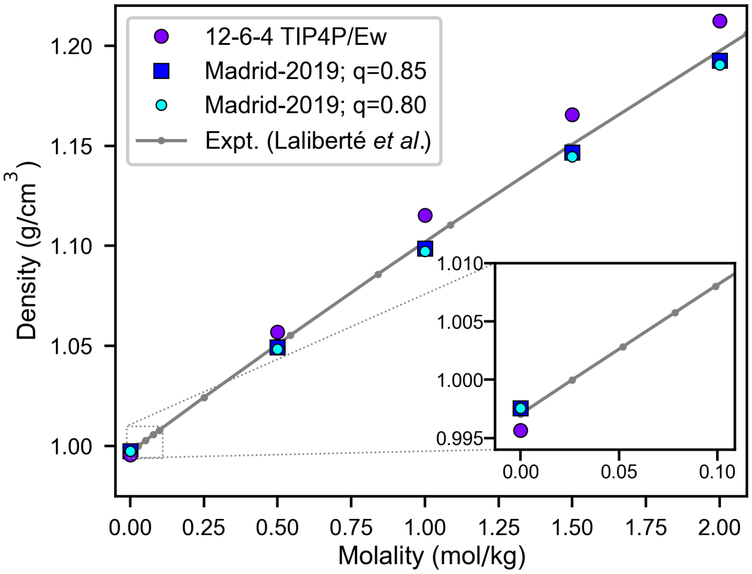

Figure S4: Densities of $FeCl_2$ obtained from simulations, for low concentrations upto 2 m, using the proposed Madrid-2019[S1] force-field for $FeCl_2$ (assuming free ions), and the 12-6-4 potential model of Ref.[S44] for Fe^{2+} and TIP4P-Ew water along with with the Joung-Cheatham parameters[S45] for chloride-water interactions (again for TIP4P-Ew). Unlike the Madrid-2019 force field, cross-interactions are obtained using the Lorentz-Berthelot rules for the 12-6-4 potential. Experimental densities in black are taken from Laliberté and Cooper[S20] The inset depicts a close-up view of the data for very low molalities, with different ranges on both axes. The Madrid model with $q = 0.80$ e (cyan circles) uses the same parameters as those of the Madrid-2019 ($q = 0.85$ e) except for the values of σ for the $Cl-O_w$ and $Mg-O_w$ interactions which are slightly different.

In Fig. S4, we present the results for the density of $FeCl_2$, obtained from simulations using the proposed Madrid-2019 force-field, for low concentrations upto 2 m, where complexes are not expected to form. We provide a comparison with a previously published model for Fe^{2+} and water as described by the TIP4P/Ew[S46] model, the 12-6-4 potential of Li and Merz Jr[S44], using Joung-Cheatham parameters[S45] for the chloride – water interactions (chloride parameters specifically designed for TIP4P-Ew) , and Lorentz-Berthelot mixing rules for cross-interactions. The densities obtained with the 12-6-4 potential show a systematic positive deviation of about 2% from the experimental densities, for concentrations up to 2 m. The agreement of the TIP4P/Ew[S46] and

TIP4P/2005[S47] water models with experimental data for the density of pure water (i.e., at zero molality) is excellent, with deviations within 0.15%, as shown in the inset. It should be mentioned that the force-field for Fe^{2+} from Ref.[S44] was designed for the cation at infinite dilution and was not tested at high concentrations of salt.

8.2 Simulations incorporating complexes

Table S3: Densities (ρ), in g/cm^3 at 298.15 K and 1 bar, of $FeCl_2$ and $MgCl_2$ at 4 m, but with varying α (percentage of free cations in the system), calculated using the Madrid-2019 force field for both Fe^{2+} and Mg^{2+}. Note that $\alpha + \alpha' = 100$, since we assume that cations are either free or that they participate in monomers.

System	α	α'	ρ
$MgCl_2$	100	0	1.248
$MgCl_2$	15	85	1.254
$MgCl_2$	2.5	97.5	1.255
$FeCl_2$	100	0	1.363
$FeCl_2$	15	85	1.369
$FeCl_2$	2.5	97.5	1.370

In this work, we also study the effect of complex speciation on the densities. We define α, α' and α'' as the percentage of free cations, monomers and dimers, respectively. Considering a mixture composed of only free cations and monomers, we compare the values of densities for a particular concentration (4 m) for both $MgCl_2$ and $FeCl_2$, as shown in Table S3. We conclude that the densities are relatively insensitive to complex formation, since the differences in densities correspond to a difference of about 0.5%. We speculate that the density change caused by the inclusion of chloride ions in octahedral solvation shells (compared to a situation in which only water is in an octahedral solvation shell) is not significant.

9 Overestimation of viscosities by Madrid-2019

The Madrid-2019 model has been known to slightly overestimate viscosities. However, we show that this overestimation cannot explain the large deviation from experimental viscosities for $MgCl_2$

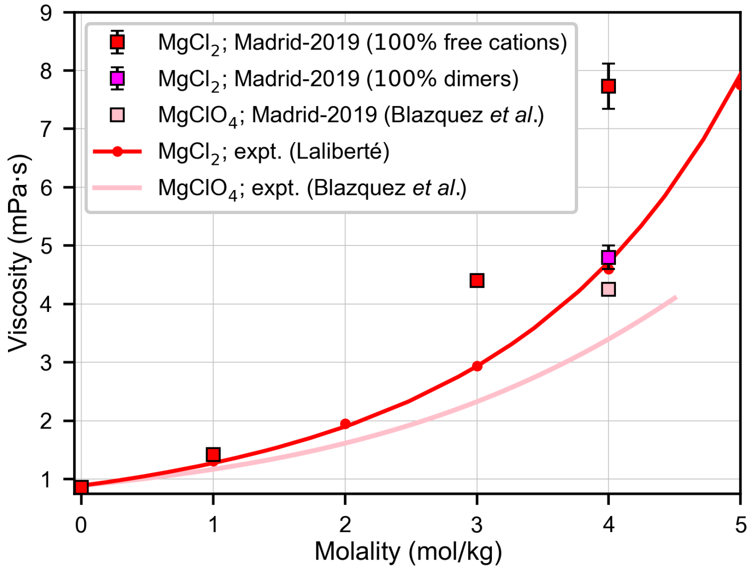

Figure S5: Viscosities of $MgCl_2$ (solid red line) and $Mg(ClO_4)_2$ from experiments (solid pink line) and from simulations with the Madrid-2019 force-field (filled red and pink squares, respectively). Simulation results at 4 m are from this work. Experimental results for $Mg(ClO_4)_2$ are from Blazquez et al.[S3] Filled magenta square: simulation results for the Madrid-2019 force field of $MgCl_2$ at 4 m assuming that all cations form $[Mg(H_2O)_4Cl_2]$.

and $FeCl_2$. For instance, in the case of NaCl, the error in the prediction, at 4 m, was ≈ 0.4 mPa·s, whereas for $MgCl_2$ it is ≈ 2.5 mPa·s. In the same vein, we observe that in the case of $Mg(ClO_4)_2$ (a solution which is somewhat similar to $MgCl_2$) shown in Fig. S5(b), the model overestimates the viscosity by about 1 mPa·s.

We argue that complex formation in $FeCl_2$ and $MgCl_2$ solutions, at higher concentrations, causes the deviations in computed viscosities (from simulations wherein all cations are free). Artificially inserting complexes (such that all cations form "dimers" or $[Mg(H_2O)_4Cl_2]$ complexes) brings the computed viscosity value closer to the experimental value, as shown in Fig. S5.

10 Speciation effects on transport properties

The viscosity and diffusion coefficient obtained in simulations for 4 m solutions of $FeCl_2$ and $MgCl_2$ are presented in Table S4.

In Table S5, we present the product of the D_{H_2O} and the viscosity of the system. This product is

Table S4: Viscosity (in mPa·s) of $FeCl_2$ and $MgCl_2$ solutions at 4 m obtained from simulations. α denotes the percentage of free cations in the system (only monomers are considered so that $\alpha + \alpha' = 100$). The Yeh-Hummer correction[S7] is applied to the diffusion coefficients to account for finite-size effects.

System	α	$D_{H_2O} \times 10^9$ (m^2/s)	η (mPa·s)
$MgCl_2$	100	0.366	7.73(0.39)
$MgCl_2$	15	0.499	5.71(0.33)
$MgCl_2$	2.5	0.522	5.56(0.42)
$FeCl_2$	100	0.369	7.68(0.41)
$FeCl_2$	15	0.496	5.87(0.30)
$FeCl_2$	2.5	0.522	5.54(0.32)

Table S5: The product of the diffusion coefficient of water, and the viscosity of the solution ($D_{H_2O} \times \eta$), reported in units of $10^{12}\times$ J/m, for $FeCl_2$ and $MgCl_2$ at 4 m. α denotes the percentage of free cations in the system. The Yeh-Hummer correction[S7] was applied to the diffusion coefficients (shown in units of m^2/s after being multiplied by 10^9) to account for finite-size effects. Note that $\alpha + \alpha' = 100$, since we assume that cations are either free or that they participate in monomers.

System	α	$D_{H_2O} \times \eta$
$MgCl_2$	100	2.8
$MgCl_2$	15	2.8
$MgCl_2$	2.5	2.9
$FeCl_2$	100	2.8
$FeCl_2$	15	2.9
$FeCl_2$	2.5	2.9

approximately constant and it is around 2.8(2) 10^{12} J/m.) This is interesting, since it shows that a rough estimate of the viscosity can be obtained from the diffusion coefficient of water (a relatively cheap calculation).

Table S6: Diffusion coefficient ($\times 10^9 m^2/s$) of the cations of $FeCl_2$ and $MgCl_2$ at 4 m using the same force field for Fe^{2+} and Mg^{2+}. Results are shown for $\alpha = 100$ (free ions, no monomers) and for $\alpha = 2.5$ (the majority of the cations is in the monomer form). Note that $\alpha + \alpha' = 100$, since we assume that cations are either free or that they participate in monomers.

	$\alpha = 100$	$\alpha = 2.5$
	$D_{M^{2+}}$	$D_{M^{2+}}$, $D_{MCl^{+}}$
$MgCl_2$	0.0955	0.15(5), 0.129(4)
$FeCl_2$	0.0922	0.15(3), 0.130(2)

The diffusion of the cations is shown in Table S6. For $\alpha = 100$, the diffusion of Fe is smaller than that of Mg (as expected from its higher mass). When monomers are formed, the diffusion coefficient of the cation increases significantly both for the free cation and for the cation forming the complex.

11 Back-of-the-envelope calculation for viscosities

Table S7: Changes in the viscosity η in mPa·s, of $MgCl_2$ at the concentration of 4 m with the degree of association. Calculations are performed with the Madrid-2019 force field using the $q = 0.8$ e model. Results are presented for free ions $\alpha = 100$, monomers $\alpha' = 100$ and dimers $\alpha'' = 100$

System	α	α'	α''	η
Free ion	100	0	0	5.33(0.3)
monomer complexes	0	100	0	4.12(0.3)
dimer complexes	0	0	100	3.90(0.3)

In the main text, we proposed a simple relation between the viscosity with 100% free ions, and the distribution of complexes (α, α' and α'')

Here, we estimate the coefficients of α' and α'' in Eq (2) in the main text, denoted by δ' and δ'', respectively (the rate of change of viscosity with α'' or α'). Therefore, for the $q = 0.85$ e and $q = 0.80$ e Madrid-2019 models, we obtain $\delta' = 0.020$ mPa $\cdot$ s and $\delta' = 0.010$ mPa $\cdot$ s, respectively, from the slopes of lines fitted to the data in Fig. 3 of the main text.

The value of δ'' for the $q = 0.85$ e model can be obtained from:

$$\begin{aligned} &\frac{\eta_{(\alpha=100,\alpha'=0,\alpha''=0)} - \eta_{(\alpha=0,\alpha'=0,\alpha''=100)}}{100} \\ =&\frac{7.73-4.80}{100}\ \text{mPa}\cdot\text{s} \\ \approx&0.029\ \text{mPa}\cdot\text{s}, \end{aligned} \tag{S3}$$

where the viscosity values were obtained for the $q = 0.85$ Madrid-2019 model.

The value of δ'' for the $q = 0.8\ e$ model can be obtained from:

$$\begin{aligned}
&\frac{\eta_{(\alpha=100,\alpha'=0,\alpha''=0)} - \eta_{(\alpha=0,\alpha'=0,\alpha''=100)}}{100} \\
=&\frac{5.33 - 3.90}{100}\ \text{mPa}\cdot\text{s} \\
\approx&0.014\ \text{mPa}\cdot\text{s},
\end{aligned} \tag{S4}$$

where the viscosity values were obtained for the $q = 0.8\ e$ Madrid-2019 model, collected in Table S7.

Furthermore, we provide justifications for the values taken for $\log K_1$ and $\log K_2$. Values of $\log K_1$) (i.e., logarithm to base 10) and $\log K_2$ for some divalent cations with many ligands can be found in the monumental and trusted Lange's Handsbook.[S48] For Fe^{2+}, the handbook only reports $\log K_1$ and refers to the value of Martell and Smith[S49] (equal to 0.36). However, the handbook does provide the values of $\log K_1$ and $\log K_2$ for Fe^{3+}, equal to 1.48 and 0.65, respectively, as well as those for Pb^{2+}, equal to 1.62 and 0.82, respectively. Of course, the individual values depend on the cation, but we observe that the approximation $\log K_2 \approx \log K_1 - 0.8$ seems reasonable. Intuitively, this makes sense since the Cl^--Cl^- electrostatic repulsion would make the formation of the second complex somewhat less favorable (that means that the second equilibrium constant is similar to the first and differs from it, at most, by an order of magnitude). In this experiment, we shall set all activity coefficients to 1 (we stress, once again, that we are just attempting to do a qualitative calculation). By assuming the value -0.20 for $\log K_1$ from NIST, and -1.0 for $\log K_2$, we obtain (for a 4 m solution of $FeCl_2$): $\alpha = 22$, $\alpha' = 56$ and $\alpha'' = 22$ (Visual MINTEQ with the SIT model, and without dimer formation included, would lead to $\alpha = 29$, which is not so different from our value). By replacing the value η^0 for the model with $q = 0.8$ (i.e 5.33 mPa·s for $\alpha = 100$), and using Eq. (6) from the main text with these values of α, α' and α'', one obtains 4.46 mPa·s for the viscosity of $FeCl_2$, which is not so different from the experimental value measured in this work (equal to 4.17 mPa·s; see Table 1 in the main text). By assuming a $\log K_1$ of -1.1 for $MgCl_2$ (i.e., 0.9 below the value of $FeCl_2$), and $\log K_2 = -1.1 - 0.8 = -1.9$ we obtain, for a 4 m solution of

$MgCl_2$: $\alpha = 64$, $\alpha' = 33$, $\alpha'' = 3$. Similarly, we obtain 4.96 mPa·s for the viscosity of $MgCl_2$, which is very close to the viscosity of $MgCl_2$ from experiments (as shown in Fig. S1).

12 Evaluation of the Jones-Dole coefficient of a force-field

The Jones-Dole equation describes the variation of the viscosity of electrolyte solutions quite well, according to the relation:

$$\eta/\eta_w = 1 + A\sqrt{m} + Bm + Cm^2 + ... \tag{S5}$$

where η is the viscosity of the solution at molality m and η_w is the viscosity of pure water. The coefficient A is always positive and rather small and is at least one order of magnitude smaller than B. The term A captures the viscosity change due to Coulombic ion-ion interactions. The term B originates from from ion-water interactions, and the term C represents the interactions between hydrated ions and the formation of complexes. Determining A and B from simulations is a *tour of force*, as it requires extremely long simulations to estimate the viscosity with very high accuracy at low concentrations. In fact, there is only one paper in the literature[S50] which rigorously evaluates the Jones-Dole coefficients for certain popular force-fields of 1:1 electrolytes. In that work, B coefficients were obtained by fitting the simulation results for concentrations between 0.1 and 1 m, while the values of A were not reported, since the associated statistical error was larger than the absolute values. In this work, we estimate the B coefficients by using the simple expression (obtained from Eq. (S5) after neglecting the quadratic term):

$$B' \simeq (\eta/\eta_w - 1 - A\sqrt{m})/m \tag{S6}$$

Given that $A << B$, we use the experimental values of A to obtain B'. The value of A is is $\approx$ 0.02 $(kg/mol)^{1/2}$ for 2:1 electrolytes.[S51] Furthermore, the results of Yue and Panagiotopoulos[S50] strongly suggest that the values of A of atomistic force-fields are very close to the experimental ones. To evaluate B', a concentration which is low enough that the quadratic term contribution in

the Jones-Dole equation (Eq. (S5)) can be neglected is needed. We shall use 0.6 m for $MgCl_2$ and $FeCl_2$. In fact, if one uses experimental results[S19] for η and η_w at 0.6 m for $CaCl_2$ and $MgCl_2$ in Eq. (S6), one obtains a value of B' that is only 0.01 kg/mol higher than the experimental value of B reported by Marcus[S51,S52] for these salts (0.01 kg/mol is also the experimental uncertainty of B). The notation B' just reflects that B' (as given by Eq. (S6)) is an approximation of the true value of B (although it is a good approximation as when implemented with experimental values of viscosities since it deviates by only 0.01 kg/mol from the exact value of B).

In order determine the coefficient B', we performed 24 independent simulations (at 0.6 m) in the *NVT* ensemble (keeping the rest of the simulation details the same as those specified in Sec. 1.1) of 20 ns each (i.e., we used a total simulation time of 480 ns to determine the viscosity of one salt and force-field). The volume used in the *NVT* simulations was obtained from a *NpT* run of 20 ns. The system contains 4440 molecules of water and the number of ions needed for the required concentration, i.e., 0.6 m. Our estimated uncertainty for B' is 0.03 kg/mol (obtained from the uncertainties of the viscosities of the solutions and that of pure water plus that introduced by Eq. (S6)). The viscosity of TIP4P/2005 water at 298.15 K and 1 bar is 0.866(3) mPa·s (the experimental one is 0.890 mPa·s). Using the force-field described in the main text for the scaled charge 0.8, we obtained (at 0.6 m and 298.15 K and 1 bar) a viscosity of 1.120(4) mPa.s for $FeCl_2$ and of 1.097(4) for $MgCl_2$. Therefore, we obtained $B' = 0.46(3)$ kg/mol for $FeCl_2$ and $B' = 0.42(3)$ kg/mol for $MgCl_2$.

References

(S1) Zeron, I.; Abascal, J.; Vega, C. A force field of Li^+, Na^+, K^+, Mg^{2+}, Ca^{2+}, Cl^-, and SO_4^{2-} in aqueous solution based on the TIP4P/2005 water model and scaled charges for the ions. *J. Chem. Phys* **2019**, *151*, 134504.

(S2) Abascal, J. L. F.; Vega, C. A general purpose model for the condensed phases of water: TIP4P/2005. *J. Chem. Phys.* **2005**, *123*, 234505.

(S3) Blazquez, S.; Troncoso, J.; La Francesca, P.; Gallo, P.; Conde, M.; Vega, C. Extending the Madrid-2019 force field to the perchlorate anion: role of charge distribution and validation with experiments on Mars-relevant aqueous solutions. *J. Mol. Liq.* **2025**, 128035.

(S4) Blazquez, S.; Conde, M.; Vega, C. Scaled charges for ions: An improvement but not the final word for modeling electrolytes in water. *J. Chem. Phys* **2023**, *158*, 054505.

(S5) Abraham, M. J.; Murtola, T.; Schulz, R.; Páll, S.; Smith, J. C.; Hess, B.; Lindahl, E. GROMACS: High performance molecular simulations through multi-level parallelism from laptops to superComput.ers. *SoftwareX* **2015**, *1–2*, 19–25.

(S6) Thompson, A. P.; Aktulga, H. M.; Berger, R.; Bolintineanu, D. S.; Brown, W. M.; Crozier, P. S.; in 't Veld, P. J.; Kohlmeyer, A.; Moore, S. G.; Nguyen, T. D.; Shan, R.; Stevens, M. J.; Tranchida, J.; Trott, C.; Plimpton, S. J. LAMMPS - a flexible simulation tool for particle-based materials modeling at the atomic, meso, and continuum scales. *Comput.. Phys. Commun.* **2022**, *271*, 108171.

(S7) Yeh, I.-C.; Hummer, G. System-Size Dependence of Diffusion Coefficients and Viscosities from Molecular Dynamics Simulations with Periodic Boundary Conditions. *J. Phys. Chem. B* **2004**, *108*, 15873–15879.

(S8) Evans, D. J.; Holian, B. L. The Nose–Hoover thermostat. *J. Chem. Phys* **1985**, *83*, 4069–4074.

(S9) Parrinello, M.; Rahman, A. Polymorphic transitions in single crystals: A new molecular dynamics method. *J. Appl. Phys* **1981**, *52*, 7182–7190.

(S10) Nosé, S.; Klein, M. Constant pressure molecular dynamics for molecular systems. *Mol. Phys.* **1983**, *50*, 1055–1076.

(S11) Hess, B.; Bekker, H.; Berendsen, H. J.; Fraaije, J. G. LINCS: A linear constraint solver for molecular simulations. *J. Comput.. Chem.* **1997**, *18*, 1463–1472.

(S12) Shinoda, W.; Shiga, M.; Mikami, M. Rapid estimation of elastic constants by molecular dynamics simulation under constant stress. *Phys. Rev. B* **2004**, *69*, 134103.

(S13) Hockney, R. W.; Eastwood, J. W. *Comput.er simulation using particles*; CRC Press: Boca Raton, 2021.

(S14) Ryckaert, J.-P.; Ciccotti, G.; Berendsen, H. J. Numerical integration of the cartesian equations of motion of a system with constraints: molecular dynamics of n-alkanes. *J. Comput.. Phys.* **1977**, *23*, 327–341.

(S15) Martínez, L.; Andrade, R.; Birgin, E. G.; Martínez, J. M. PACKMOL: A package for building initial configurations for molecular dynamics simulations. *J. Comput.. Chem.* **2009**, *30*, 2157–2164.

(S16) Jewett, A. I.; Stelter, D.; Lambert, J.; Saladi, S. M.; Roscioni, O. M.; Ricci, M.; Autin, L.; Maritan, M.; Bashusqeh, S. M.; Keyes, T.; Dame, R. T.; Shea, J.-E.; Jensen, G. J.; Goodsell, D. S. Moltemplate: A Tool for Coarse-Grained Modeling of Complex Biological Matter and Soft Condensed Matter Physics. *J. Mol. Biol* **2021**, *433*, 166841.

(S17) Hjorth Larsen, A.; Jørgen Mortensen, J.; Blomqvist, J.; Castelli, I. E.; Christensen, R.; Dułak, M.; Friis, J.; Groves, M. N.; Hammer, B.; Hargus, C.; Hermes, E. D.; Jennings, P. C.; Bjerre Jensen, P.; Kermode, J.; Kitchin, J. R.; Leonhard Kolsbjerg, E.; Kubal, J.; Kaasbjerg, K.; Lysgaard, S.; Bergmann Maronsson, J.; Maxson, T.; Olsen, T.; Pastewka, L.; Peterson, A.; Rostgaard, C.; Schiøtz, J.; Schütt, O.; Strange, M.; Thygesen, K. S.; Vegge, T.; Vilhelmsen, L.; Walter, M.; Zeng, Z.; Jacobsen, K. W. The atomic simulation environment—a Python library for working with atoms. *J. Phys. Condens. Matter* **2017**, *29*, 273002.

(S18) Gonzalez, M. A.; Abascal, J. L. F. The shear viscosity of rigid water models. *J. Chem. Phys* **2010**, *132*, 096101.

(S19) Laliberte, M. Model for calculating the viscosity of aqueous solutions. *J. Chem. Eng. Data* **2007**, *52*, 321–335.

(S20) Laliberté, M.; Cooper, W. E. Model for calculating the density of aqueous electrolyte solutions. *J. Chem. Eng. Data* **2004**, *49*, 1141–1151.

(S21) Königsberger, E.; Königsberger, L.-C.; Szilágyi, I.; May, P. M. Measurement and Prediction of Physicochemical Properties of Liquors Relevant to the Sulfate Process for Titania Production. 1. Densities in the $TiOSO_4 + FeSO_4 + H_2SO_4 + H_2O$ System. *J. Chem. Eng. Data* **2008**, *54*, 520–525.

(S22) Uroš Luin, U.; Arcon, I.; Valant, M. Structure and Population of Complex Ionic Species in $FeCl_2$ Aqueous Solution by X-ray Absorption Spectroscopy. *Molecules* **2022**, *27*, 642.

(S23) Ohtaki, H.; Radnai, T. Structure and dynamics of hydrated ions. *Chem. Rev.* **1993**, *93*, 1157–1204.

(S24) Marcus, Y. Ionic radii in aqueous solutions. *Chem. Rev.* **1988**, *88*, 1475–1498.

(S25) Marcus, Y. Thermodynamics of solvation of ions. Part 5.—Gibbs free energy of hydration at 298.15 K. *J. Chem. Soc. Faraday Trans.* **1991**, *87*, 2995–2999.

(S26) Persson, I. Hydrated metal ions in aqueous solution: How regular are their structures? *Pure Appl. Chem.* **2010**, *82*, 1901–1917.

(S27) Zapałowski, M.; Bartczak, W. M. Concentrated aqueous $MgCl_2$ solutions. A Comput.er simulation study of the solution structure and excess electron localisation. *Res. Chem. Intermed.* **2001**, *27*, 855–866.

(S28) Schmidt, H.; Hennings, E.; Voigt, W. Magnesium chloride tetrahydrate, $MgCl2 \cdot 4H_2O$. *Acta Crystallogr. C* **2011**, *68*, i4–i6.

(S29) Lind, M. D. Crystal Structure of Ferric Chloride Hexahydrate. *J. Chem. Phys* **1967**, *47*, 990–993.

(S30) Kuppuraj, G.; Dudev, M.; Lim, C. Factors governing metal- ligand distances and coordination geometries of metal complexes. *J. Phys. Chem. B* **2009**, *113*, 2952–2960.

(S31) Wei, Z.; Xu, H.; Xu, X.; Feng, G.; Zheng, W.; Li, T. Solvation of magnesium chloride dimer in water: The case of anionic and neutral clusters. *J. Chem. Phys* **2023**, *158*, 174311.

(S32) Wróbel, P.; Kubisiak, P.; Eilmes, A. Quantum-Chemical and Molecular Dynamics Investigations of Magnesium Chloride Complexes in Dimethoxyethane Solutions. *ACS Omega* **2020**, *5*, 12842–12852.

(S33) Shi, R.; Cooper, A. J.; Tanaka, H. Impact of hierarchical water dipole orderings on the dynamics of aqueous salt solutions. *Nature Communications* **2023**, *14*.

(S34) Chaudhari, M. I.; Vanegas, J. M.; Pratt, L.; Muralidharan, A.; Rempe, S. B. Hydration mimicry by membrane ion channels. *Annu. Rev. Phys. Chem* **2020**, *71*, 461–484.

(S35) Karsten Meier, K.; Laesecke, A.; Kabelac, S. Transport coefficients of the Lennard-Jones model fluid. I Viscosity. *J. Chem. Phys* **2004**, *121*, 3671.

(S36) Gustafsson, J. P. Visual MINTEQ 3.0 user guide. *KTH, Department of Land and Water Recources, Stockholm, Sweden* **2011**, *550*.

(S37) Bronsted, J. N. Studies on solubility IV. The principle of the specific interaction of ions. *J. Am. Chem. Soc.* **1922**, *44*, 877.

(S38) Guggenheim, E.; Turgeon, J. Specific interaction of ions. *Trans. Faraday Soc.* **1955**, *51*, 747.

(S39) Ciavatta, L. The specific interaction theory in the evaluating ionic equilibria. *Trans. Faraday Soc.* **1980**, *70*, 551.

(S40) Davies, C. W. 397. The extent of dissociation of salts in water. Part VIII. An equation for the mean ionic activity coefficient of an electrolyte in water, and a revision of the dissociation constants of some sulphates. *J. Chem. Soc.* **1938**, 2093.

(S41) Duboué-Dijon, E.; Mason, P. E.; Fischer, H. E.; Jungwirth, P. Hydration and Ion Pairing in Aqueous Mg^{2+} and Zn^{2+} Solutions: Force-Field Description Aided by Neutron Scattering

Experiments and Ab Initio Molecular Dynamics Simulations. *J. Phys. Chem. B* **2017**, *122*, 3296–3306.

(S42) Böhm, F.; Sharma, V.; Schwaab, G.; Havenith, M. The low frequency modes of solvated ions and ion pairs in aqueous electrolyte solutions: iron(II) and iron(III) chloride. *Phys. Chem. Chem. Phys* **2015**, *17*, 19582–19591.

(S43) Luin, U.; Arčon, I.; Valant, M. Structure and Population of Complex Ionic Species in $FeCl_2$ Aqueous Solution by X-ray Absorption Spectroscopy. *Molecules* **2022**, *27*, 642.

(S44) Li, P.; Merz Jr, K. M. Taking into account the ion-induced dipole interaction in the non-bonded model of ions. *J. Chem. Theory Comput.* **2014**, *10*, 289–297.

(S45) Joung, I. S.; Cheatham III, T. E. Determination of alkali and halide monovalent ion parameters for use in explicitly solvated biomolecular simulations. *J. Phys. Chem. B* **2008**, *112*, 9020–9041.

(S46) Horn, H. W.; Swope, W. C.; Pitera, J. W.; Madura, J. D.; Dick, T. J.; Hura, G. L.; Head-Gordon, T. Development of an improved four-site water model for biomolecular simulations: TIP4P-Ew. *J. Chem. Phys* **2004**, *120*, 9665–9678.

(S47) Abascal, J. L. F.; Vega, C. A general purpose model for the condensed phases of water: TIP4P/2005. *J. Chem. Phys* **2005**, *123*, 234505.

(S48) Dean, J. A. *Lange's Handbook of Chemistry, 15th Edition*; McGraw-Hill: New York, 1999.

(S49) Martell, A. E.; Smith, R. M. *Critical stability constants*; Springer, 1974; Vol. 1.

(S50) Yue, S.; Panagiotopoulos, A. Z. Dynamic properties of aqueous electrolyte solutions from non-polarisable, polarisable, and scaled-charge models. *Mol. Phys.* **2019**, *117*, 3538–3549.

(S51) Jenkins, H. D. B.; Marcus, Y. Viscosity B-Coefficients of Ions in Solution. *Chem.Rev.* **1995**, *95*, 2695.

(S52) Marcus, Y. *Ion Properties*; Marcel Dekker, Inc., 1997.
